# Selection of sites for field trials of genetically engineered mosquitoes with gene drive

**DOI:** 10.1111/eva.13283

**Published:** 2021-08-10

**Authors:** Gregory C. Lanzaro, Melina Campos, Marc Crepeau, Anthony Cornel, Abram Estrada, Hans Gripkey, Ziad Haddad, Ana Kormos, Steven Palomares

**Affiliations:** ^1^ Vector Genetics Laboratory Department of Pathology, Microbiology and Immunology School of Veterinary Medicine University of California Davis California USA; ^2^ California Institute of Technology Jet Propulsion Laboratory Pasadena California USA

**Keywords:** *Anopheles gambiae*, genetic control, islands, malaria, population modification

## Abstract

Novel malaria control strategies using genetically engineered mosquitoes (GEMs) are on the horizon. Population modification is one approach wherein mosquitoes are engineered with genes rendering them refractory to the malaria parasite, *Plasmodium falciparum*, coupled with a low‐threshold, Cas9‐based gene drive. When released into a wild vector population, GEMs preferentially transmit these parasite‐blocking genes to their offspring, ultimately modifying a vector population into a nonvector one. Deploying this technology awaits ecologically contained field trial evaluations. Here, we consider a process for site selection, the first critical step in designing a trial. Our goal is to identify a site that maximizes prospects for success, minimizes risk, and serves as a fair, valid, and convincing test of efficacy and impacts of a GEM product intended for large‐scale deployment in Africa. We base site selection on geographic, geological, and biological, rather than social or legal, criteria. We recognize the latter as critically important but not as a first step in selecting a site. We propose physical islands as being the best candidates for a GEM field trial and present an evaluation of 22 African islands. We consider geographic and genetic isolation, biological complexity, island size, and topography and identify two island groups that satisfy key criteria for ideal GEM field trial sites.

## 1 INTRODUCTION

We present a framework employed by the University of California Irvine Malaria Initiative (UCIMI) for the selection of sites to conduct field trials of a genetically engineered mosquito (GEM) with gene drive. These GEMs are designed to offer safe, cost‐effective, and sustainable malaria control in sub‐Saharan Africa. This will be achieved using a population modification strategy (Carballar‐Lejarazú & James, 2017) wherein parasite‐blocking effector genes are engineered into vector mosquitoes, rendering them incapable of transmitting the parasite (Isaacs et al., 2012). An essential GEM component is a highly efficient gene drive (Carballar‐Lejarazú et al., 2020). These threshold‐independent drives will spread through a population even when introduced at very low frequencies, thereby serving two critical purposes: to establish the effector genes at high frequency in the mosquito population at the immediate release site and to facilitate its spread into neighboring populations via normal mosquito dispersal and gene flow. This GEM is designed to eliminate the malaria parasite, *P*. *falciparum* without eliminating the mosquito.

Achieving malaria control on a large spatial scale requires a threshold‐independent gene drive, meaning one with a maximum capability for spreading across the environment (invasiveness). Henceforth, when we refer to a GEM, we mean a mosquito engineered with anti‐*Plasmodium* effector genes and a threshold‐independent, highly invasive gene drive. This is the GEM that UCIMI aims to evaluate in a field trial.

Our primary goal for a site selection process is identification of a site that maximizes the prospects for success, minimizes risk, and serves as a fair, valid, and convincing test of the efficacy and impacts of a GEM product. The UCIMI program targets the principal malaria vectors, *Anopheles coluzzii* and *A*. *gambiae*, and is ultimately intended for malaria elimination throughout sub‐Saharan Africa. Early field trials will provide critically important data to inform subsequent trials and influence decisions regarding large‐scale deployment. These data include factors that cannot easily be assessed in caged populations because cages do not adequately replicate natural conditions and cannot assess phenomenon on a meaningful spatial scale. We consider a trial successful when the quality and utility of the data it generates justifies the time, effort, and cost that goes into conducting it. Phase 2 field trials are intended to measure entomological endpoints including GEM survival, mating competitiveness, gene drive spread, and construct stability (WHO/TDR & FNIH, 2014). Biological complexity can complicate data interpretation. Therefore, selection of a site with minimal complexity will maximize the prospects for success. Identifying field sites with hard boundaries that prevent gene flow into and out of the site results in a high level of containment. Containment minimizes the risk of GEM invasion outside the target site and maximizes the prospects for success by minimizing immigration of wild‐type individuals into the study population. For a trial to be considered fair, it is our opinion that it be conducted at a site that is well justified on scientific grounds. To be valid and convincing, a trial should generate data that define key parameters accurately and results should contribute to assessing GEM performance over a range of environmental conditions. In the following narrative, we set forth a framework describing how these goals may be achieved.

A multi‐phase pathway for the development and evaluation of GEMs has been proposed by the World Health Organization (WHO; WHO/TDR & FNIH, 2014). This protocol has been widely endorsed (African Union & NEPAD, 2018; National Academies of Sciences Engineering & Medicine, 2016) and serves as the foundation for the framework described here. PHASE 1 of the WHO pathway includes design and construction of the GEM product and initial evaluation of its efficacy. This evaluation assesses the phenotype generated by the transgenes, transgene inheritance (especially as it relates to the efficiency of the gene drive component), the stability of the construct over time, and a rudimentary evaluation of overall fitness (Rebeca Carballar‐Lejarazú et al., 2020; Hammond et al., 2016). GEM products that show promise then move into PHASE 2 field trials with a strong emphasis on containment.

Early guidelines recommended that initial tests be conducted in large, artificially contained greenhouse‐like cages designed to simulate natural conditions (Alphey et al., 2002; Benedict et al., 2008; Facchinelli et al., 2013; Scott et al., 2002). Data generated in such caged environments are limited in several important ways: They do not allow analysis of community and ecosystem‐level interactions in any meaningful sense, they cannot replicate food web structure, and they do not permit examination of ecological phenomenon (e.g., dispersal) across spatial scales (Carpenter, 1996; Wynn & Paradise, 2001). Critically, experiments conducted in artificial environments often yield highly replicable, but spurious results (Schindler, 1998). These limitations were recognized in later guidelines and the use of artificially contained environments is now suggested as optional, unless required by regulatory authorities (James et al., 2018, 2020).

A different strategy that has been proposed for dealing with containment is to conduct field trials in a stepwise fashion with early trials using threshold‐dependent drives. A threshold‐dependent drive will only spread within a population when introduced above some threshold frequency. Examples include split‐drive systems which have limited invasiveness and are therefore self‐contained (Cisnetto & Barlow, 2020; Nash et al., 2019). Threshold‐dependent drives have their place in controlling vectors on a small spatial scale, such as in urban settings (Li et al., 2020); however, deploying a high‐threshold drive to achieve malaria control at the scale of continental Africa is not feasible (James et al., 2018).

From our perspective, conducting trials in large cages or with high‐threshold drives does not satisfy our goal that field tests be valid and convincing. Therefore, we propose to use ecologically confined PHASE 2 field trials in their place. The issue of containment can be mitigated by selecting the appropriate site.

The first consideration in the selection of a GEM field site should be based on defining biological and physical characteristics that would make a site ideal, or as near to ideal, as possible (Alphey et al., 2002; Knols & Bossin, 2006; Scott et al., 2002). Ethical, social, and legal issues are critically important, and no field test can be undertaken before these are addressed (Kolopack & Lavery, 2017; Neuhaus, 2018; Resnik, 2014). The UCIMI recognizes this and has adopted the relationship‐based model for community and regulatory engagement which we have described in detail in a recent publication (Kormos et al., 2020). However, valuable resources, relationships, and infrastructure are best developed at a site that has first been determined to be scientifically suitable. Here, we describe a set of criteria that may be applied to a thoughtful consideration and assessment of potential field trial sites. When completed, this framework should provide a cogent justification for why a particular site was selected for GEM testing.

Ecologically confined field sites should offer geographic, environmental, and/or biological confinement (WHO/TDR & FNIH, 2014). Ecological islands not bounded by water have been suggested as a possibility, but these are not well known for African anopheline species. Physical islands have been suggested as ideal for conducting GEM field trials (National Academies of Sciences Engineering & Medicine, 2016; Scott et al., 2002). Islands have served as model ecosystems and have played a key role in the development of evolutionary theory (Warren et al., 2015). In addition to containment, islands have numerous characteristics that favor their use as GEM field trial sites, including relatively small size, distinct boundaries, simplified biotas, and relative geological youth. These features led to the development of contemporary "Island Biogeography Theory," IBT (Frankham, 1997; Losos & Ricklefs, 2009; MacArthur & Wilson, 1967; Santos et al., 2016), which we rely on to inform our assessment of the advantages of island over mainland sites for the evaluation of GEM. Under IBT, island size (area) and geographic isolation are considered the most important factors driving island biodiversity (Helmus & Behm, 2020), and since this information is readily obtained from published sources, it formed the basis for initial selection of potential sites.

## 2 MATERIALS AND METHODS

### 2.1 Selection of candidate island sites

**FIGURE 1:**
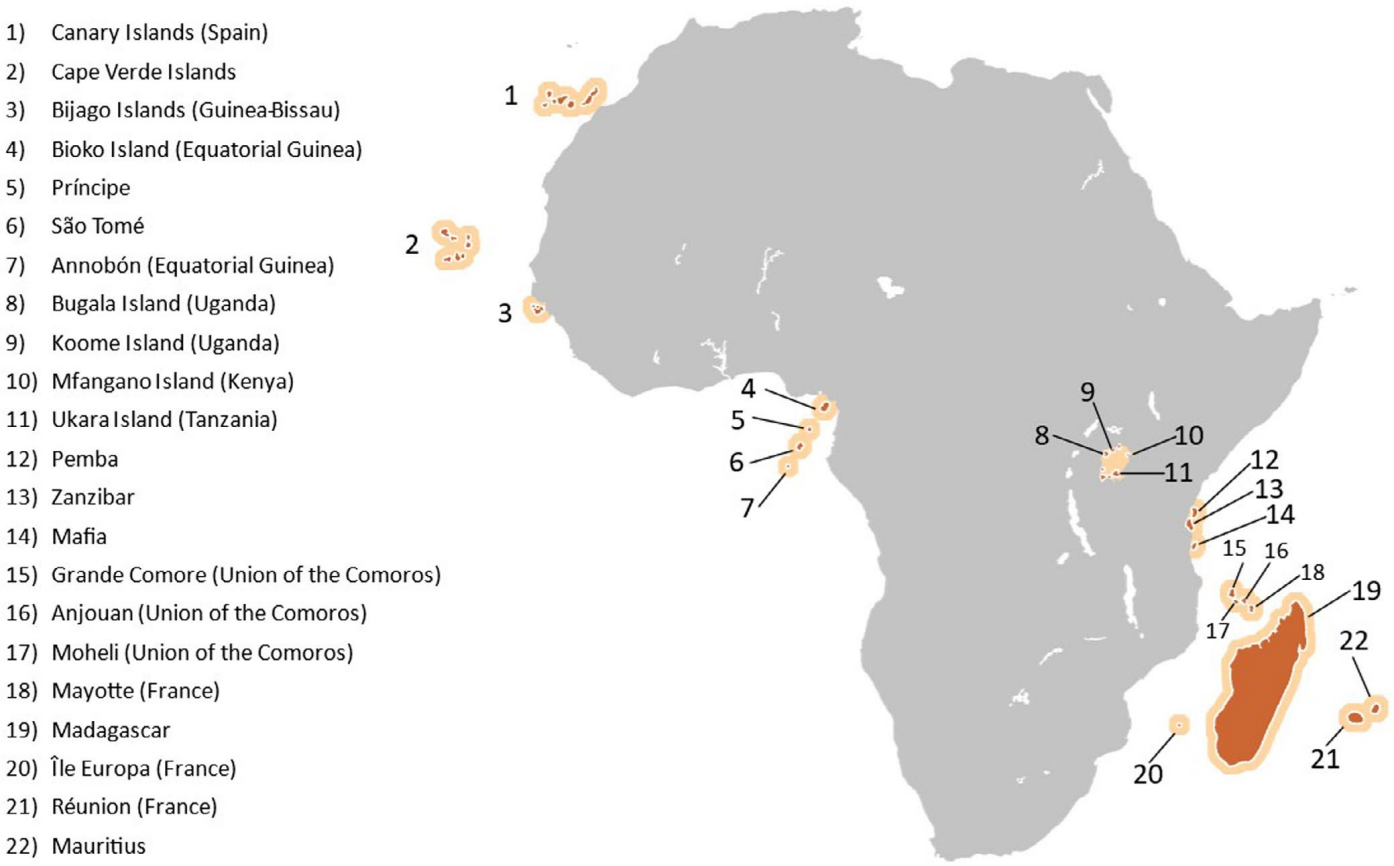
African islands and island groups considered potential field sites for genetically engineered mosquitoes for malaria eradication

Site selection was initiated with the identification of all potential island sites, which we define broadly as any island associated with the continent of Africa (Figure 1). Data for each site were obtained from published sources except for some genetic data which was generated de novo by us. These data were used to inform the suitability of potential sites by determining if they meet the set of criteria listed in Box 1. This information includes descriptions of entomological, genetic, geographic, and geophysical features of the sites and mosquito populations therein.

BOX 1 **Criteria for the selection of field sites.**

**Table:** 

**Primary criteria**	**Rationale**
1. Presence of *Anopheles gambiae*/*A. coluzzii*	Major vector; target of GEM production
2. Geographic isolation	Containment of GEMs
3. Genetic isolation	Containment of transgene constructs
4. Genetic diversity	Potential detriment to GEM function
5. Island size	Feasibility vs. validity
6. Topography	Evaluation of GEM dispersal capacity
7. Anopheline species richness	Logistics; HGT*; confound epi. endpoints
**Other considerations**	**Rationale**
1. Insecticide susceptibility/resistance	Match GEM to indigenous mosquitoes
2. *Plasmodium* prevalence	Estimation of epidemiological impact
3. Presence of endangered species	Potential for negative GEM interactions
4. Travel feasibility	Operational logistics and cost

Abbreviation: HGT, horizontal gene transfer.

### 2.2 Measuring island geographic isolation

Geographic isolation for each island was defined using three methods, all reported in Table 1.

**TABLE 1:** Bioclimatic and isolation index values used for the evaluation of potential island field sites

Island	Archipelago	Island type	Area (km²)	Distance to mainland (km)	UNEP Isolation Index	SLMP	GMMC	Elevation max (m)
Canary Islands	Canary Islands	Oceanic	7509.66	116.63	30.4	0.812	0	3705
Cape Verde	Cape Verde Islands	Oceanic	4088.52	586.53	55	0.466	0	2813
Bijagós	Bijagós Islands	Continental	1944.72	0.83	10.8	1.123	1	59
Bioko	Cameroon Line	Continental	1950.46	73.03	17	1.148	1	3011
Annobón	Cameroon Line	Oceanic	15.7	350	45	–	0	587
São Tomé	Cameroon Line	Oceanic	854.8	283.63	39	0.753	0	1977
Príncipe	Cameroon Line	Oceanic	143.16	221.72	39	0.86	0	934
Bugala	Lake Victoria	Lacustrine	296	3.7	5.487	–	1	160
Koome	Lake Victoria	Lacustrine	100	14.3	10.688	–	1	180
Mfangano	Lake Victoria	Lacustrine	66	7.4	10.414	–	1	551
Ukara	Lake Victoria	Lacustrine	80	22.8	15.623	–	1	162
Pemba	Zanzibar	Continental	987.08	68.6	31.231	1.178	0	149
Zanzibar	Zanzibar	Continental	1591.5	50.9	17	1.337	1	133
Mafia	Mafia	Continental	443.24	36.06	29.432	1.194	1	66
Grande Comore	Comoros	Oceanic	1021.61	307.45	49	0.736	0	2368
Moheli	Comoros	Oceanic	212.09	340.61	49	0.754	0	793
Anjouan	Comoros	Oceanic	432.08	417.78	49	0.706	0	1591
Mayotte	Comoros	Oceanic	371.42	490.16	47	0.669	0	636
Madagascar	Madagascar	Oceanic	590,547.4	780.51	58	0.46	0	2876
Ile Europa	French Territory	Oceanic	32.64	492.95	67.941	0.736	0	20
Réunion	Mascarene Islands	Oceanic	2512.65	1699.32	73	0.467	0	3066
Mauritius	Mascarene Islands	Oceanic	1868.44	1874.49	87	0.399	0	816

Note

DD, decimal degrees; UNEP, United Nations Environment Programme; SLMP, surrounding landmass proportion; GMMC, glacial maximum mainland connection, a proxy variable for island geological history which indicates whether an island was connected to the mainland during the last glacial maximum (LGM; 1 = true and 0 = false); – refers to missing or incomplete data. Additional data sources: Bugala:(Kayondo et al., 2005; Nambuya et al., 2013; Ssegawa & Nkuutu, 2006; Zeemeijer, 2012); Mfango (Idris et al., 2016); Ukara: (Lounio, 2014; Mugono et al., 2014; Smith, 1955); and Koome (BakamaNume, 2010; Google Earth; Jackson & Gartlan, 1965; Nampijja et al., 2015; Tuhebwe et al., 2015).

The first metric is simply the geographic distance to the nearest mainland. Distances for individual islands were calculated as the shortest great circular distance between an island's mass centroid and the mainland coast. For archipelagos, distances from the nearest island to the mainland were used (Weigelt et al., 2013). Distance to the mainland for each Lake Victoria island and for Annobón was determined using Google Earth's distance and area measuring tool. The two closest points on the mainland and island shores were used as measuring points. The significance of distance to mainland is that the nearest mainland is assumed to be the richest gene pool and the source of populations on the islands (Itescu et al., 2020; Weigelt et al., 2013).

A second metric is the United Nations Environment Programme (UNEP) Isolation Index, which is calculated as "the sum of the square roots of the distances to the nearest equivalent or larger island, the nearest group or archipelago, and the nearest continent (Dahl, 1991).” The higher the value, the more geographically isolated the island is.

The third isolation index is surrounding landmass proportion (SLMP) where the isolation of the focal island is proportional to the area of the surrounding landmass (Weigelt et al., 2013). SLMP is calculated as the sum of the proportions of landmass within buffer distances of 100, 1000, and 10,000 km around the island perimeter. SLMP accounts for the coastline shape of large landmasses by considering only regions that extend into the measured buffers. SLMP values for the Canary Islands, Cape Verde Islands, and Bijagós Islands were represented as the average of all islands in their respective archipelagos (Weigelt et al., 2013). SLMP is a preferred index for analysis of species variation on a focal island. The equilibrium theory of island biogeography supports this index as individual islands may act as stepping stones for species dispersal and establishment, which this index accounts for by shortening the distance between an island and potential source populations (MacArthur & Wilson, 1967). A larger SLMP value indicates that an island is surrounded by more landmass. For this study, we are focusing on islands with a lower SLMP value since these islands will have less surrounding landmass which could facilitate mosquito dispersal into or out of the target island.

### 2.3 Island size and topography

Island size information, presented in Table 1 as area, is taken from the publication by Weigelt et al. (2013). They describe island size by using the Database of Global Administrative Areas (GADM) to obtain high‐resolution island polygons. Area was calculated for each GADM polygon in a cylindrical equal area projection. Areas for archipelagos (Canary Islands, Bijagós, Cape Verde) were reported here as the sum of all islands in each archipelago (Weigelt et al., 2013). The area for Annobón was obtained from the United Nations Environmental Programme (Dahl, 1991). The areas for the Lake Victoria islands (excluding Koome) were taken from the literature (Idris et al., 2016; Lounio, 2014; Zeemeijer, 2012), and the area for Koome Island was approximated using Google Earth's distance and area measuring tool.

Elevation maximum and minimum of each island were obtained from the AW3D30 Global Digital Surface Model of the Japan Aerospace Exploration Agency (Japan Aerospace Exploration Agency, 2020). GeoTIFF files were downloaded, and the highest elevation of each island/archipelago was identified. Island topography was further described using the United States National Aeronautics and Space Administration (NASA) 90m resolution elevation data from the Shuttle Radar Topography Mission (SRTM) 90m Digital Elevation Model database (Jarvis et al., 2008). In this case, altitude and magnitude of steepest gradient measurements were used to generate heat maps as graphic descriptors of topography.

### 2.4 Population genomics analyses

**FIGURE 2:**
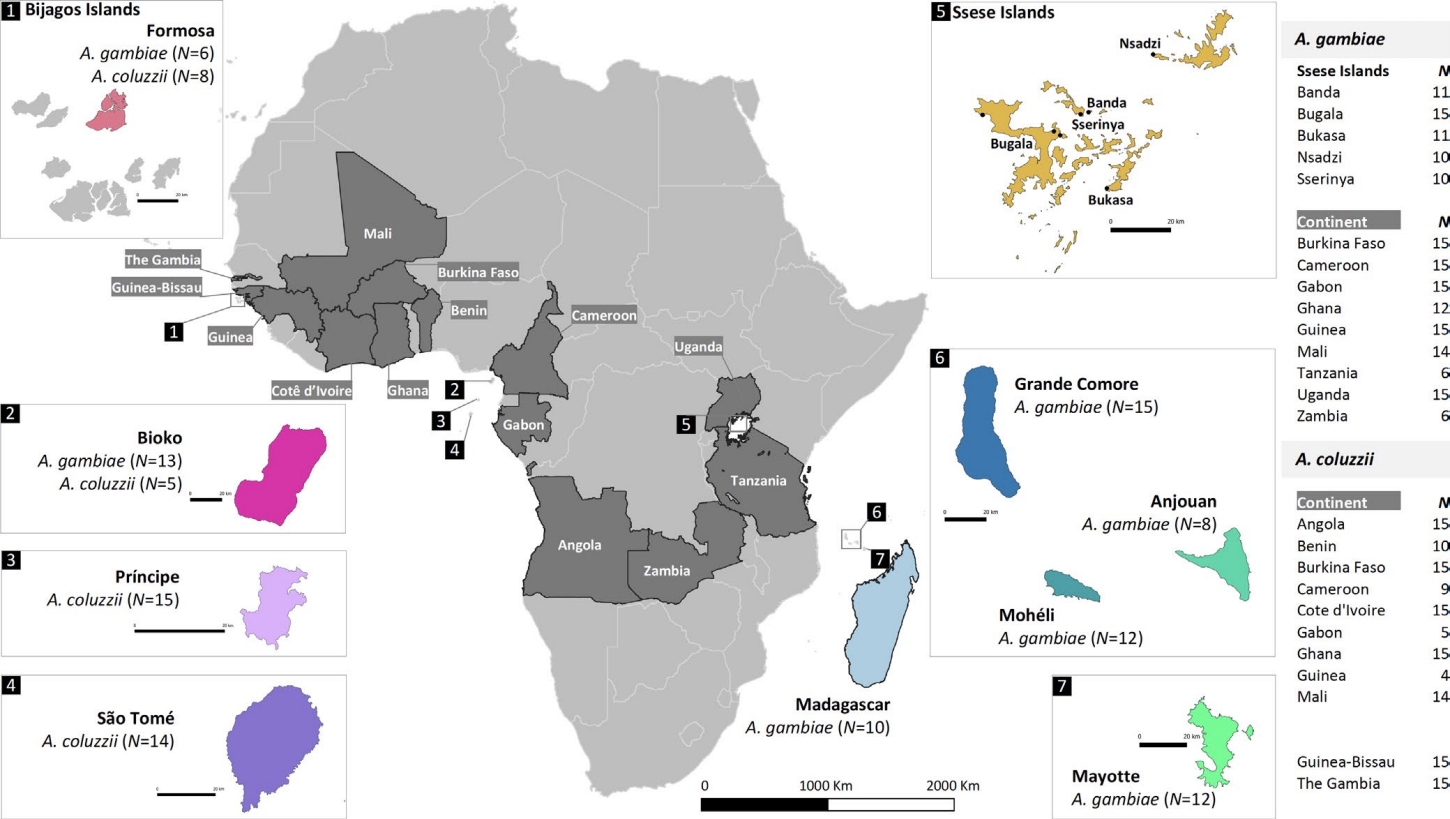
Study sampling locations. Samples of *Anopheles gambiae* and *Anopheles coluzzii* were from 12 countries (dark shade) in continental Africa: Angola, Benin, Burkina Faso, Cameroon, Cotê d’Ivoire, Gabon, Ghana, Guinea, Tanzania, Uganda, and Zambia. Populations from Guinea‐Bissau and The Gambia (dark shade) were included with no species assignment. Table on the right displays the number of samples for each of the mainland populations. The insert maps show African islands sampled in this study: (1) Formosa islands within Bijagos archipelago; (2) Bioko, (3) Príncipe and São Tomé, (4) islands in the Gulf of Guinea, (5) five islands in Ssese islands in Lake Victoria in Uganda, (6) in the Comoros (Anjouan, Mohéli and Grande Comore), and (7) Mayotte. Madagascar Island is shown in the main map. The number of samples included for each island is shown in parentheses. Insert maps contain a scale of 20 km length

We conducted a comparative genomics analysis of mainland and island populations of the two target species. The locations and sample sizes per site are provided in Figure 2. In total, 420 individual *Anopheles gambiae* and *A*. *coluzzii* genome sequences were analyzed in this study. The UC Davis Vector Genetics Laboratory (VGL) generated 167 genomes (Table S1). In addition, 196 genomes were obtained from the *Anopheles gambiae* 1000 Genome Project phase 2 (Anopheles gambiae, 1000 Genomes Consortium, 2020) and 57 were taken from a published Lake Victoria islands study (Bergey et al., 2020).

Individual mosquito DNAs from the VGL samples were extracted using a Qiagen Biosprint (Qiagen) following our established protocol (Nieman et al., 2015). 10 ng of genomic DNA was used for individual libraries using a KAPA HyperPlus Kit (Roche Sequencing Solutions), as described in Yamasaki et al. (2016). Sequencing was performed on an Illumina HiSeq 4000 instrument (Illumina) at the UC Davis DNA Technologies Core facility. Methods used for genome sequencing of individuals from other sources are described elsewhere (Bergey et al., 2020; Clarkson et al., 2021).

Demultiplexed raw reads of VGL samples were filtered and trimmed using Trimmomatic v0.36 (Bolger et al., 2014) and saved as FastQ files. Sequences from Ag1000G and Lake Victoria study (Bergey et al., 2020) were downloaded and converted to FastQ files using BEDTools v.2.2 (Quinlan & Hall, 2010). All specimens were mapped to the reference AgamP4 (Holt et al., 2002; Sharakhova et al., 2007) using BWA‐MEM v0.7.15 (Li, 2013) with default settings. Duplicate reads were removed using Sambamba markdup (Tarasov et al., 2015). Freebayes v1.2.0 (Garrison & Marth, 2012) was used for variant calling, with standard filters and the “‐no‐population‐priors,” “theta = 0.01,” and “max‐comple‐gap = 0” options. Variants were normalized with *vt normalize* v0.5 (Tan et al., 2015).

Single nucleotide polymorphisms were filtered out when they did not pass the accessibility mask from Ag1000G, missingness >10%, a minimum depth of 8 and minor allele frequency (MAF) <1%. In addition, population structure analysis was based on chromosome 3 SNPs only. This was done to avoid confounding signals from polymorphic inversions on chromosomes 2 and X (Sharakhova et al., 2007). Heterochromatic regions on chromosome 3R (3R:38,988,757–41,860,198; 3R:52,161,877–53,200,684) and 3L (3L:1–1,815,119; 3L:4,264,713–5,031,692) were also filtered out (Sharakhova et al., 2007).

Description of population structure was performed by principal component analysis (PCA) after pruning for LD using scikit‐allel v1.2.0 (Miles & Harding, 2017). Hudson's estimator (Bhatia et al., 2013; Hudson et al., 1992) was used for pairwise fixation indices *F*_ST_ calculation implemented in scikit‐allel v1.2.0. Nucleotide diversity (π) was calculated in nonoverlapping windows of 10 kb on euchromatic regions of chromosome 3 using VCFtools (Danecek et al., 2011). The results were grouped by population and significance tests performed between the islands and mainland populations using a Wilcoxon rank‐sum test in R.

### 2.5 Anthropogenic sources of dispersal

The prospects for a GEM emigrating out of a field trial site into a nontarget site or vice versa by air or ship transport were assessed by determining the frequency of departures from select mainland and island sites. Airline flight data including the annual (Jan. 1–Dec. 31, 2019) number of international departures from airports within a specific country were obtained from CIRIUM, an aviation data analytics provider (LNRS Data Services Inc, 2021). Similarly, marine shipping data for the annual (Jan. 1–Dec. 31, 2020) number of commercial ship departures were obtained from the (Exmile Solutions Ltd, 2021).

### 2.6 Anopheline species richness

Published compilations of Afrotropical *Anopheles* species distributions (Irish et al., 2020; Kyalo et al., 2017) were used to assemble the information for mainland and island countries. The first criterion for field site selection is the presence of the target species, which is in our case *Anopheles gambiae* sensu stricto and/or its sister species *Anopheles coluzzii*. Species are designated as primary or secondary vectors or as “other” if they are nonvectors or their status as vectors is not clear. Species that commonly had sporozoite infection rates above 1%, as determined by salivary gland dissections, CSP ELISA or PCR of head and thorax were listed as primary vectors. Species with infection rates of <1% were listed as secondary vectors. Our knowledge of the population structure and biology of almost all the secondary vectors is limited, and their role in malaria transmission varies from location to location.

## 3 RESULTS AND DISCUSSION

### 3.1 Identification of potential field sites

We evaluated 22 potential island field sites, including 5 individual islands, multiple islands within 7 archipelagos, and 4 islands within Lake Victoria (Figure 1). The sites identified include three island types: continental, oceanic, and lacustrine. Each type possesses features that impact its utility as a GEM trial site. Continental or land‐bridge islands are unsubmerged portions of the continental shelf and were, at one time, connected to the mainland. Oceanic islands arise from the ocean floor and were never connected to the mainland. Lacustrine islands are islands within lakes and are typically formed by deposits of sedimentary rock, as are the Lake Victoria islands. For comparison, our analyses include mainland sites closest to the islands and those in which GEM field trials are currently under consideration (e.g., Burkina Faso, Mali, Uganda). We then proceed by defining and justifying a prioritized set of criteria (Box 1) on which to base evaluations.

### 3.2 Geographic isolation

Geographic isolation is among the most significant features favoring islands as GEM field trial sites. Although some mosquito species are known to disperse on prevailing winds over long distances (Huestis et al., 2019; Services, 1997), there are, to our knowledge, no reliable reports of open‐ocean wind dispersal of malaria vector species over the distances (hundreds of kilometers) separating some of the oceanic islands under consideration here. Emigration of GEMs out of the field trial site into neighboring, nontarget sites, either on nearby islands or on the mainland, poses a problem, especially as it relates to risk and regulatory concerns. Equally important is immigration of wild‐type individuals from neighboring sites into the trial site. Immigration, in this case, will confound efforts to measure GEM invasiveness and could potentially render the gene drive inefficient or even ineffective. Island biogeography theory predicts that choosing a remote island as an initial field trial site greatly reduces the potential for gene flow between vector populations both into and out of the island site. This is further supported by the results of our population genomics assessment, as discussed below.

We evaluated geographic isolation for all candidate islands using distance to mainland, UNEP Isolation Index, and SLMP (Table 1). We excluded any island with a UNEP Isolation Index of <15 and we used a surrounding landmass proportion (SLMP) value of 1 as a cutoff, so islands with an SLMP value >1 were considered unacceptable. Sites considered unacceptable based on these criteria include the Bijagos Islands, Bugala, Koome, Mfangano, Pemba, Zanzibar, and Mafia.

### 3.3 Island size and topography

There are no well‐defined criteria to guide decisions with respect to an appropriately sized area for a GEM field trial. One important consideration is mosquito flight range. To evaluate the dispersal capacity of a GEM, the site should exceed the flight range of the target species. For our considerations, we assumed a maximal daily flight range of 10 km for *A*. *gambiae* (Kaufmann & Briegel, 2004). Generally, we aimed to identify sites small enough to be manageable, but large enough to be convincing, keeping the following considerations as a guide.

Area (km²) is an important parameter influencing the biology of populations residing on an island. Large island areas typically include more habitat types and can support larger populations. This characteristic can increase the rate of speciation and lower extinction rates over time (Santos et al., 2016). Using island size as a criterion, we exclude the islands of Annobón and Île Europa for being too small and Madagascar for being too large.

Evaluating the dispersal capabilities of a GEM is a critical outcome from a field trial. This capacity is best evaluated at a site that possess topographical features that may pose a challenge to dispersal, as would be encountered in continental Africa. Elevation was used as a measure of topographic complexity and as a proxy for environmental heterogeneity. The difference between the elevation maximum and minimum of each island measured from sea level is reported in the “Elevation” column in Table 1. Elevation relates to the number of available habitats because of differences between windward and leeward sites, temperature decrease with altitude, and high precipitation regimes at certain altitudes (Weigelt et al., 2013).

Altitude and magnitude of steepest gradient were used to generate a graphic representation of topography for each island. A representative sample of these analyses for the islands of Grande Comore and São Tomé is presented in Figure S1a,b to illustrate sites having the desired level of topographic complexity and for the islands of Zanzibar and Mafia in Figure S1c,d to illustrate a lack of suitable topographic features. Sites lacking topographic complexity were excluded from consideration; these included the Bijagos Islands, the islands in Lake Victoria, Zanzibar, Pemba, Mafia, and Ile Europa.

### 3.4 Genetic isolation

Genetic isolation relates to the level of gene flow between populations and may be inferred by measuring the degree of genetic divergence between populations under the assumption that gene flow reduces genetic divergence.

Single nucleotide polymorphism data were analyzed to reveal genetic relationships among populations, and results were visualized using principal component analysis (PCA). The position of individuals in the space defined by the principal components can be interpreted as revealing levels of genetic similarity/dissimilarity among the populations from which those individuals were sampled. Populations occupying the same space are presumed to be very similar genetically and those widely separated, very different.

**FIGURE 3:**
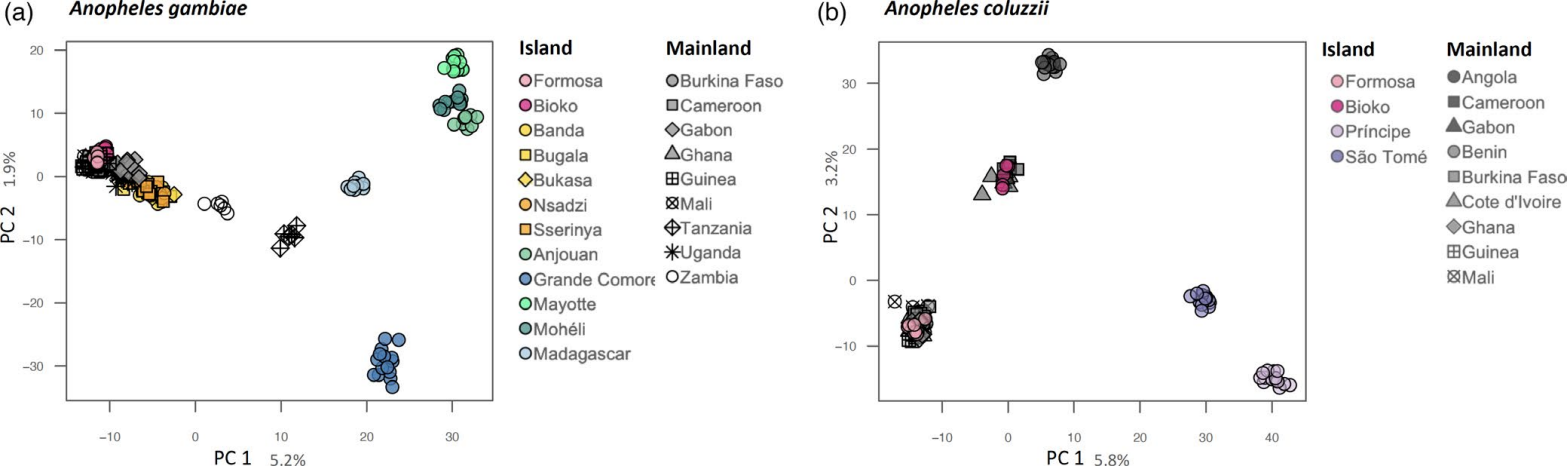
Population structure analysis by PCA. 2D‐plot of *Anopheles gambiae* (a) and *Anopheles coluzzii* (b) from islands and mainland populations across Africa. Analyses were based on 50,000 biallelic SNPs from euchromatic regions on chromosome 3. Each marker represents one individual mosquito. Geographic location for each site and numbers of genome analyzed per site are provided in Figure 2

Results of the PCA for *A*. *gambiae* populations are illustrated in Figure 3a. This analysis reveals a high degree of genetic similarity between mainland and both lacustrine and continental islands. Conversely, oceanic islands (Comoros archipelago and Madagascar) form discrete individual clusters, indicating that they are genetically distinct both from the mainland and from each other. Results of the PCA for *A*. *coluzzii* (Figure 3b) confirm that populations on continental islands form tight clusters that include mainland populations. In contrast, oceanic islands form discrete clusters indicating genetic divergence from mainland populations and from each other. These results indicate high levels of genetic isolation for oceanic island populations of both *A*. *coluzzii* and *A*. *gambiae*.

The extent to which individuals move (migrate) between two populations can be approximated by measuring the level of genetic divergence between those populations. Migration (*m*) can be thought of as including the genotypes of the individuals doing the moving and, in this context, migration results in gene flow. Genetic divergence can be described using the statistic *F*_ST_, which is the genetic variance in a subpopulation (S) relative to the total variance (T). *F*_ST_ values range between 0 and 1 and are higher when populations are considerably diverged. The relationship between *F*_ST_ and *m* is complex, but excluding the effects of drift and selection, the more gene flow between two populations, the lower the *F*_ST_ value. All pairwise *F*_ST_ values for the populations of *A*. *gambiae* and *A*. *coluzzii* analyzed in this study (Figure 2) are presented in Figure S2.

Pairwise *F*_ST_ values for 14 populations of *A*. *gambiae* spanning its range across sub‐Saharan Africa are provided in Figure S2a. Results are consistent with the PCA (Figure 3a). West‐central populations, including the island of Formosa in the Bijagos archipelago, are very similar (*F*_ST_ = 0.000–0.009). Divergence between Bioko island and the nearest mainland in Cameroon is higher (*F*_ST_ = 0.036). The pattern is quite different in east Africa, wherein mainland populations are far more diverged (*F*_ST_ = 0.033–0.090). This pattern is consistent with the theory that *A*. *gambiae* originated in west Africa and dispersed eastward through a series of population bottlenecks (Schmidt et al., 2019) Divergence between the islands in Lake Victoria and the nearest mainland in Uganda is lower (*F*_ST_ = 0.003–0.029). Considerably higher divergence is observed between the Comoros islands and the nearest mainland populations in Tanzania (*F*_ST_ = 0.130–0.169) and between the Comoros and Madagascar (*F*_ST_ = 0.126–0.196).

The *F*_ST_ values for populations of *A*. *coluzzii* are likewise consistent with the PCA (Figure 3b). Divergence between the continental island of Formosa and the nearest mainland populations in Guinea‐Bissau and between the island of Bioko and nearest sites in Cameroon is low (*F*_ST_ = 0.015 and 0.022, respectively). Populations of *A*. *coluzzii* on the oceanic islands of São Tomé and Príncipe were, by far, the most genetically isolated from mainland populations (*F*_ST_ = 0.144 and 0.199, respectively). In addition, the two islands were highly diverged from each other (*F*_ST_ = 0.130). The islands within Lake Victoria were excluded from consideration because the *A*. *gambiae* populations residing on them lacked the high level of divergence that would indicate genetic isolation.

**FIGURE 4:**
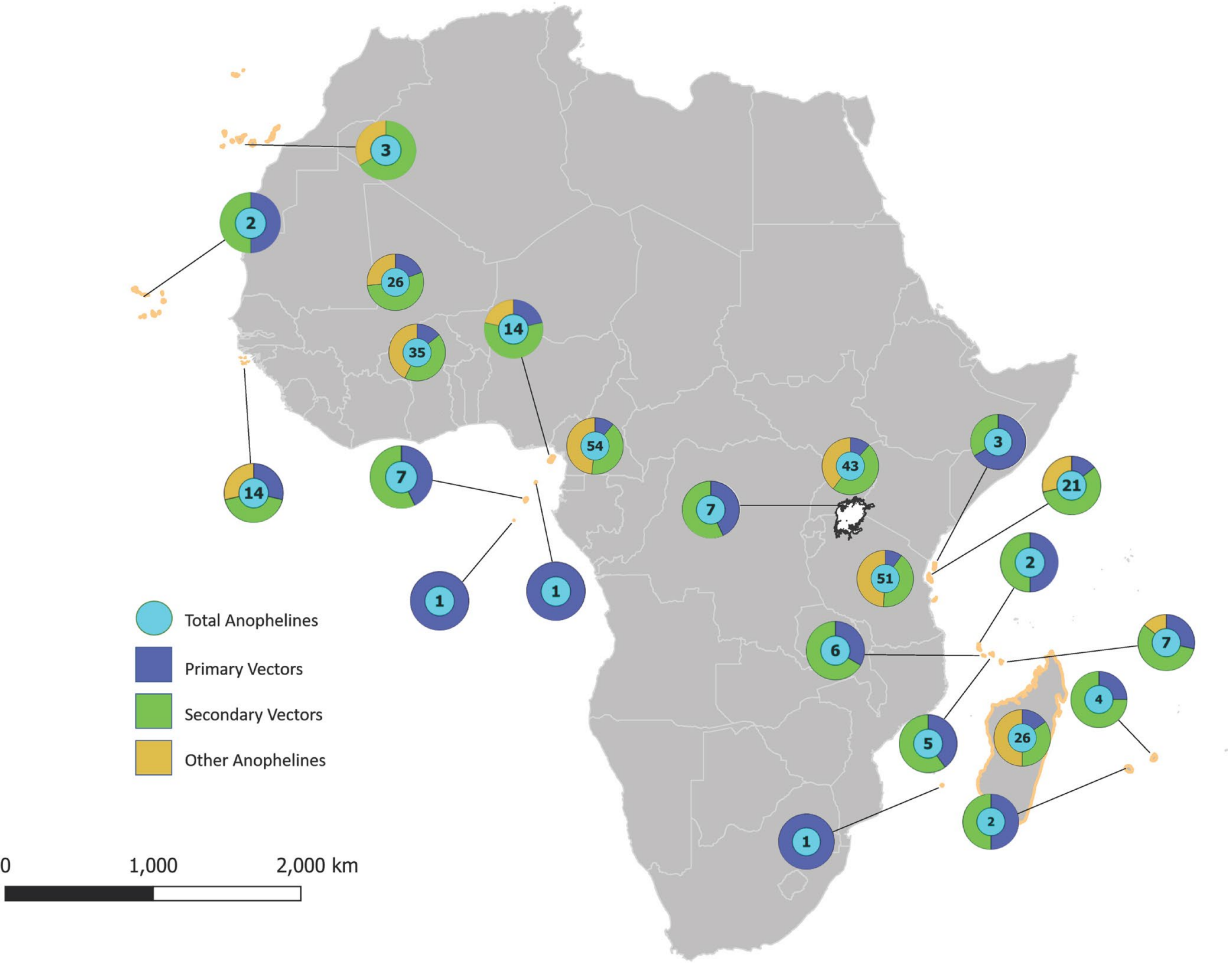
*Anopheles* species complexity in Africa including island and select mainland sites. Map locations and summary of data presented in Table S2. Cyan circle = total number of *Anopheles* spp.; blue proportion of primary vector species; green = proportion of secondary vectors; and yellow = proportion of species identified as nonvector or for which vector status unknown

Taken together, the data summarized in Figure 4 and Figure S2 reveal a high degree of genetic isolation among oceanic islands compared with either continental or lacustrine islands. These results indicate limited dispersal (gene flow) between islands and nearest landmasses, are consistent with expectations based on IBT as described above, and reinforce the benefits of selecting a contained island site for conducting GEM field trials. Genetic data are not currently available for several potential island sites, including the Canary Islands, Cape Verde, Île Europa, Zanzibar, Pemba, and Mafia. Genetic isolation, as measured here, was deemed inadequate for the Lake Victoria islands (Bugala, Koome, Mfangano, and Ukara).

### 3.5 Anthropogenic dispersal

Anthropogenic dispersal of mosquitoes from inside the release site into nontarget populations and vice versa may occur and should be considered in selecting a field trial site. The level of genetic divergence between island and mainland populations of *A*. *coluzzii* and *A*. *gambiae* is generally high suggesting that dispersal off the islands is low. Nonetheless, dispersal that may occur is most likely to rely on anthropogenic conveyance (Belkin, 1962; Services, 1997).

The most significant source of passive anthropogenic dispersal of mosquitoes is by rail and road. This poses significant risk for mainland field sites, where extensive in‐country and trans‐boundary connections exist (Campos et al., 1961; Eritja et al., 2017; Frean et al., 2014). Risk by this mode of mosquito dispersal is reduced to zero for oceanic island test sites.

**FIGURE 5:**
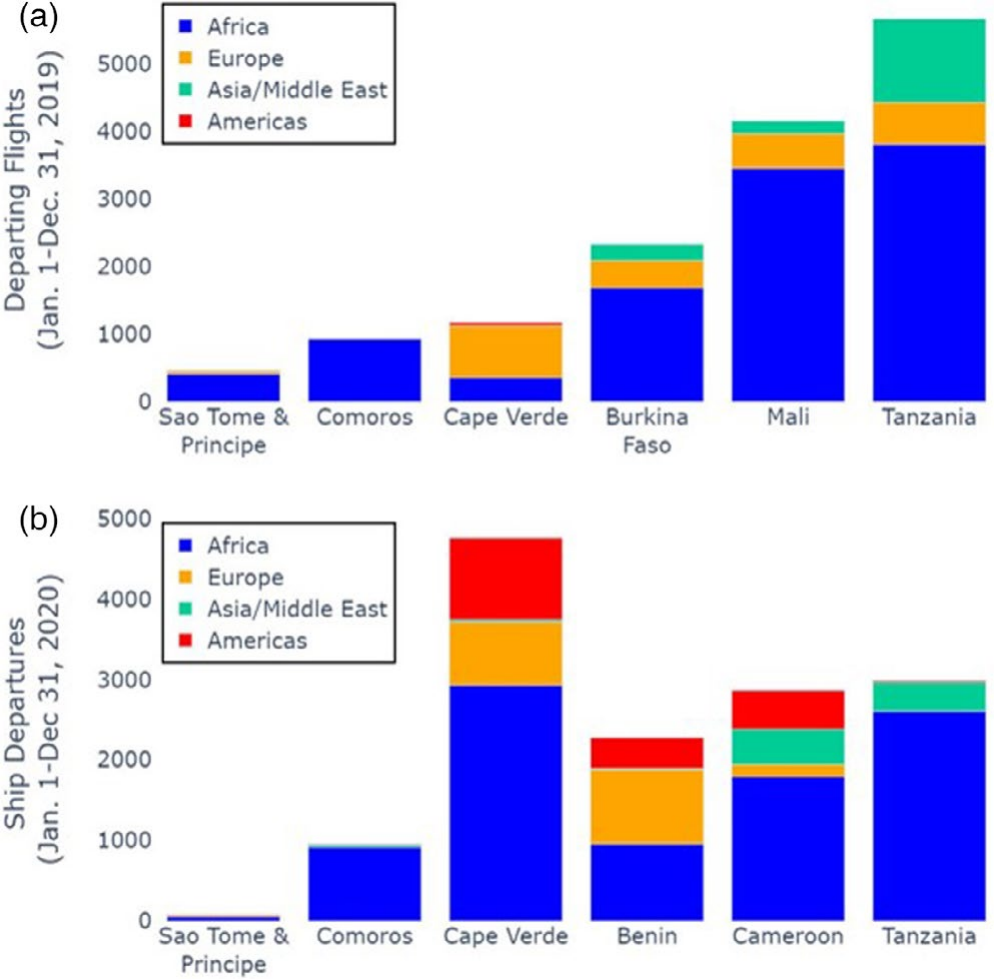
Annual departures by air (a) and sea (b) from representative island and mainland locations in Africa. Colors indicate destinations, grouped by geographic region. Air traffic data provided by Cirium*. *This information has been extracted from a Cirium product. Cirium has not seen or reviewed any conclusions, recommendations, or other views that may appear in this document. Cirium makes no warrantees, express or implied, as to the accuracy, adequacy, timeliness, or completeness of its data or its fitness for any particular purpose. Cirium disclaims any and all liability relating to or arising out of use of its data and other content or to the fullest extent permissible by law. Sea traffic data provided by the MarineTraffic Global Ship Tracking Intelligence database

Frequency of air and sea departure to interim and final destinations for a sample of mainland and island populations is presented in Figure 5 and Tables S3 and S4. Islands, due to their smaller human populations and geographic areas, generally originate less trans‐boundary air and sea traffic compared with the continent (Figure 5a). This results in remote islands that are least connected by shipping having inherently lower risk levels for these modes of anthropogenic dispersal (Helmus et al., 2014). A notable exception is the Cape Verde archipelago which has relatively high ship travel due to its location as a major refueling site (Figure 5b). Traffic has increased with the completion of two new ports and upgrades to existing ports in 1997. Airline and shipping traffic data were only obtained for the locations shown in Figure 5; therefore, assessment of the potential for anthropogenic dispersal for the majority of island sites was not assessed. Results for São Tomé and Príncipe and for the Comoros suggest that the likelihood of mosquitoes migrating via anthropogenic means into or out of these islands is minimal.

### 3.6 Anopheline species richness

The number of primary, secondary, and other (malaria vector status unclear) species present on island sites and select locations on the mainland are illustrated in Figure 4 (and Table S2). It is generally agreed that potential field sites with the fewest number of nontarget *Anopheles* species are desirable (Brown et al., 2014; James et al., 2020). If multiple sister species or unrecognized mating demes are present, there exists the possibility that the transgene will move between species via natural hybridization (Lee et al., 2013; White, 1971) which could add an additional level of complexity to postrelease assessments. Although the movement of transgene elements between malaria vector species may be considered desirable, it raises the specter of horizontal transfer, which is generally identified as a risk to this technology (Courtier‐Orgogozo et al., 2020).

Assessment of entomological endpoints following a GEM release requires repeated mosquito collections to quantify changes in the ratio of GEM to wild‐type mosquitoes. This necessitates sorting large numbers of individual field‐collected mosquito samples to separate target from nontarget species. For members of sibling species complexes, which are morphologically indistinguishable, this requires the application of PCR‐based diagnostics to each individual specimen. If collections include larvae, time‐consuming microscopic examination or individual PCR assays to identify species is required even for those species distinguishable morphologically in the adult stage. Logistically these procedures are greatly simplified where fewer nontarget *Anopheles* species are present, positively impacting the time and resources required for successful assessment.

Although entomological endpoints are the main consideration in evaluating the outcome of a PHASE 2 trial, epidemiological impacts should be considered where feasible. If epidemiological endpoints are to be assessed, the presence of multiple primary and secondary vectors is problematic as they can lengthen the season of malaria transmission (Antonio‐Nkondjio et al., 2006). Therefore, their presence can mask the effects that GEMs might have on transmission at a field site by maintaining the rate of transmission, even if the parasite is not present in the target mosquito species. If a site is selected in which very few malaria vector species occur, it becomes more likely that the GEM release will have a measurable impact on the level of malaria transmission.

The number of anopheline species present in the mainland sites included here ranged from 26 to 54. Continental island sites (Bijagos Islands, Bioko and Zanzibar) had between 14 and 21 species. As expected, oceanic islands contained far fewer, ranging from 1 to 7 species. These results favor the selection of the oceanic islands, São Tomé and Príncipe, Annobón, and the Comoros, for field trials. The oceanic islands including the Canary Islands, Cape Verde, Mauritius, and Reunion likewise had low numbers of anopheline species, but these were excluded because our target species *A*. *coluzzii* and/or *A*. *gambiae* are absent from these islands.

### 3.7 Genetic complexity

**FIGURE 6:**
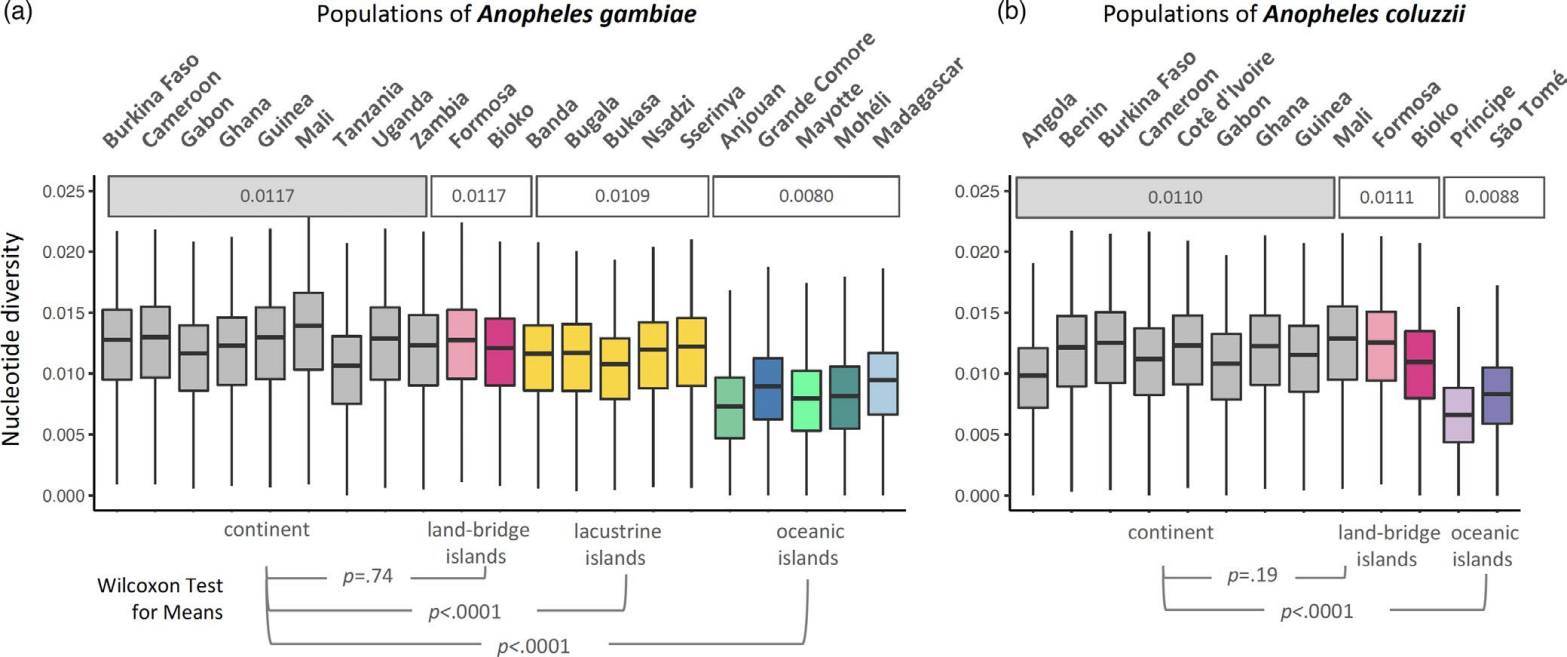
Population diversity. Metric is grouped by sampling locations of (a) *Anopheles gambiae* and (b) *Anopheles coluzzii* populations from island and mainland (gray boxplots). Boxplot of nucleotide diversity (π) performed in 10 kb windows of euchromatic regions of chromosome 3. The midline in all boxplots represents the median, with upper (75th percentile) and lower (25th percentile) limits, whiskers show maximum and minimum values, and outliers are not shown. Mean nucleotide diversity for set of populations is shown above the boxplots; *A. gambiae* populations were divided into four groups: mainland continental (gray), land‐bridge (pink), lacustrine (yellow), and oceanic (green/blue) islands and *A. coluzzii* into three: mainland continental (gray), land‐bridge (pink), and oceanic (blue) islands. *p*‐value for testing of means between islands and mainland is shown below. Geographic location for each site and numbers of genome analyzed per site are provided in Figure 2

Genetic complexity was measured using the nucleotide diversity statistic (π), defined as the average number of pairwise nucleotide differences per nucleotide site. Mean nucleotide diversities (π) on oceanic islands (*A*. *gambiae*‐ 0.80%, *A*. *coluzzii*‐ 0.88%) were significantly lower (*p* < 0.0001) than mainland population means (*A*. *gambiae*‐ 1.17%, *A*. *coluzzii*‐ 1.11%) for both species (Figure 6a,b). Comparisons among island types yielded results that were consistent with island biogeography theory. Nucleotide diversity in continental island populations did not differ from mainland populations, and lacustrine islands had only slightly lower, but statistically significant, values for π. These observations are expected given the geological history and proximity of continental and lacustrine islands to the coast. Anjouan island populations presented the lowest (0.73%) nucleotide diversity (π) for *A*. *gambiae* and Príncipe island for *A*. *coluzzii* (0.66%), likely due their small size and high degree of isolation.

In general, the lower biocomplexity on isolated islands includes reduced genetic variation (Frankham, 1997). Our results are concordant with this observation (Figure 6). Selecting field sites with populations containing the lowest levels of variation should decrease the potential for transgene/genome interactions that might negatively impact GEM performance. These include São Tomé and Príncipe and the Comoros.

### 3.8 Selection of candidate field sites

Each potential site was evaluated based on the criteria listed in Box 1. Evaluations were based on information available from the literature or calculated by us as summarized in the narrative above. Sites that fail to meet all primary criteria were eliminated from further consideration. Those sites that met all primary criteria were raised from potential status to candidate status. Some criteria require further analysis or site visits before a final evaluation can be completed. Site visits are recommended for candidate sites only. Evaluation of insecticide resistance should be conducted during site visits. Security at candidate sites is dynamic and should be evaluated regularly before and during trials. Evaluation of potential impacts on endangered species requires knowledge about the extent to which these overlap ecologically with *A*. *coluzzii* and/or *A*. *gambiae* which can only be thoroughly evaluated by mosquito collections made proximal to endangered species populations during early site visits. Initial stakeholder engagement and the establishment of collaborative partnerships during early site visits are essential for the success of the final evaluation and analysis of candidate sites.

Overall evaluations are presented in Box 2.

BOX 2 **Overall summary of evaluation of potential island sites.**

**Table:** 

	Primary criteria							Other considerations			
Island	*A. coluzzii*/*A. gambiae* present	Geographic isolation	Genetic Isolation	Genetic diversity	Size (area)	Topography	Anopheline species richness	Insecticide resistance	Plasmodium prevalence	Endangered species	Travel feasibility
Oceanic Islands											
Canary Islands	✗	✓	‐	‐	✓	✓	✓	*	✗	*	✓
Cape Verde	✗	✓	‐	‐	✓	✓	✓	*	✗	*	✓
Annobón	✓	✓	✓	‐	✗	✓	✓	*	✓	*	✗
São Tomé	✓	✓	✓	✓	✓	✓	✓	*	✓	*	✓
Príncipe	✓	✓	✓	✓	✓	✓	✓	*	✓	*	✓
Grande Comore	✓	✓	✓	✓	✓	✓	✓	*	✓	*	✓
Moheli	✓	✓	✓	✓	✓	✓	✓	*	✓	*	✓
Anjouan	✓	✓	✓	✓	✓	✓	✓	*	✓	*	✓
Mayotte	✓	✓	✓	✓	✓	✓	✓	*	✓	*	✓
Ile Europa	‐	✓	‐	‐	✗	✗	✓	*	‐	*	✗
Madagascar	✓	✓	✓	✓	✗	✓	✗	*	✓	*	✓
Mauritius	✗	✓	‐	‐	✓	✓	✓	*	✗	*	✓
Réunion	✗	✓	‐	‐	✓	✓	✓	*	✗	*	✓
Continental Islands											
Bijagós	✓	✗	✓	✗	✓	✗	✗	*	✓	*	✓
Bioko	✓	✓	✓	‐	✓	✓	✗	*	✓	*	✓
Zanzibar	✓	✗	‐	‐	✓	✗	✗	*	✓	*	✓
Pemba	✓	✗	‐	‐	✓	✗	✓	*	✓	*	✓
Mafia	✓	✗	‐	‐	✓	✗	‐	*	✓	*	✓
Lacustrine Islands (Lake Victoria)											
Bugala	✓	✗	✗	✗	✓	✗	✓	*	✓	*	✓
Koome	✓	✗	✗	✗	✓	✗	✓	*	✓	*	✓
Mfangano	✓	✗	✗	✗	✓	✗	✓	*	✓	*	✓
Ukara	✓	✗	✗	✗	✓	✗	✓	*	✓	*	✓

✓ = site meets criterion; ✗ = site fails to meet criterion; **‐** = data not available; * = to be determined when site is visited.

Evaluation of all twenty‐two potential field sites indicates that Bioko, São Tomé & Príncipe, and the Comoros Islands (Anjouan, Grande Comore, Mayotte, and Moheli) can be elevated from “potential” to “candidate” GEM field trial sites. The Mascarene (Mauritius and Réunion) and Cape Verde Islands fit many criteria, but *Anopheles gambiae* or *A*. *coluzzii* do not occur in these islands. Annobón scores well based on several of our criteria but travel there was determined to be infeasible, and the island was deemed too small to represent a trial which would provide compelling outcomes.

Therefore, we propose the following as the lead candidate sites for a PHASE 2 GEM field trial: the Comoros Islands, São Tomé and Príncipe, and Bioko.

## 4 CONCLUSIONS

Our early decision to consider physical islands as the ideal sites for a GEM field trial was guided by contemporary IBT. This theory provides the basis for certain expectations concerning species richness, in our case, anopheline species richness and also features such as genetic isolation and diversity. Consistent with IBT, anopheline mosquito species richness was lowest on small, isolated oceanic islands, higher on continental islands, and highest at mainland continental sites (Figure 5). Our results likewise confirm IBT predictions regarding relationships between geographic isolation and both genetic divergence and genetic diversity (Johnson et al., 2000) which are significantly correlated (Figure S3).

The framework described here has been applied by the University of California Irvine Malaria Initiative (UCIMI) as they enter PHASE 2 of GEM research. It is our belief that this comprehensive framework provides identification of site(s) that will maximize the prospect for success, minimize risk, and will serve as a fair, valid, and convincing test of the efficacy and impacts of the UCIMI GEM product, meeting the goal of a PHASE 2 field trial. Furthermore, this process provides a well‐reasoned, science‐based justification for selecting these sites for GEM field trials and a solid foundation on which to approach ethical, social, and legal considerations with field site stakeholders.

## CONFLICT OF INTEREST

The authors declare no conflict of interests.

## Supporting information

Fig S1

Fig S2

Fig S3

Supplementary Material

Table S1

Table S2

Table S3

Table S4

Supplementary Material

## Data Availability

The data that support the findings of this study are openly available in NCBI GenBank at https://www.ncbi.nlm.nih.gov/genbank/. New whole‐genome sequence data included in this study are under BioProject ID PRJNA729913. Sequencing read data from previous studies are available under different BioProject IDs (PRJNA607000, PRJNA648422, PRJNA590708, PRJNA433010, PRJNA493853, PRJEB1670). Accession number of each sample is provided in a supplementary table.

## References

[eva13283-bib-0001] African Union and NEPAD . (2018). Gene Drives for Malaria Control and Elimination in Africa. https://www.nepad.org/publication/gene‐drives‐malaria‐control‐and‐elimination‐africa

[eva13283-bib-0002] Alphey, L., Beard, C. B., Billingsley, P., Coetzee, M., Crisanti, A., Curtis, C., Eggleston, P., Godfray, C., Hemingway, J., Jacobs‐Lorena, M., James, A. A., Kafatos, F. C., Mukwaya, L. G., Paton, M., Powell, J. R., Schneider, W., Scott, T. W., Sina, B., Sinden, R., … Collins, F. H. (2002). Malaria control with genetically manipulated insect vectors. Science, 298(5591), 119–121. 10.1126/science.1078278 12364786

[eva13283-bib-0003] Anopheles gambiae 1000 Genomes Consortium . (2020). Genome variation and population structure among 1142 mosquitoes of the African malaria vector species *Anopheles gambiae* and *Anopheles coluzzii* . Genome Research, 30, 1–14. 10.1101/gr.262790.120 32989001 PMC7605271

[eva13283-bib-0004] Antonio‐Nkondjio, C., Kerah, C. H., Simard, F., Awono‐Ambene, P., Chouaibou, M., Tchuinkam, T., & Fontenille, D. (2006). Complexity of the malaria vectorial system in Cameroon: Contribution of secondary vectors to malaria transmission. Journal of Medical Entomology, 43(6), 1215–1221. https://www.ncbi.nlm.nih.gov/pubmed/17162956 17162956 10.1603/0022-2585(2006)43[1215:cotmvs]2.0.co;2

[eva13283-bib-0005] BakamaNume, B. B. (2010). A contemporary geography of Uganda. Mkuki Na Nyota.

[eva13283-bib-0006] Belkin, J. N. (1962). The mosquitoes of the South Pacific: (Diptera, Culicidae). University of California Press.

[eva13283-bib-0007] Benedict, M., D'Abbs, P., Dobson, S., Gottlieb, M., Harrington, L., Higgs, S., James, A., James, S., Knols, B., Lavery, J., O'Neill, S., Scott, T., Takken, W., & Toure, Y. (2008). Guidance for contained field trials of vector mosquitoes engineered to contain a gene drive system: Recommendations of a scientific working group. Vector Borne and Zoonotic Diseases, 8(2), 127–166. 10.1089/vbz.2007.0273 18452399

[eva13283-bib-0008] Bergey, C. M., Lukindu, M., Wiltshire, R. M., Fontaine, M. C., Kayondo, J. K., & Besansky, N. J. (2020). Assessing connectivity despite high diversity in island populations of a malaria mosquito. Evolutionary Applications, 13(2), 417–431. 10.1111/eva.12878 31993086 PMC6976967

[eva13283-bib-0009] Bhatia, G., Patterson, N., Sankararaman, S., & Price, A. L. (2013). Estimating and interpreting FST: The impact of rare variants. Genome Research, 23(9), 1514–1521. 10.1101/gr.154831.113 23861382 PMC3759727

[eva13283-bib-0010] Bolger, A. M., Lohse, M., & Usadel, B. (2014). Trimmomatic: A flexible trimmer for Illumina sequence data. Bioinformatics, 30(15), 2114–2120. 10.1093/bioinformatics/btu170 24695404 PMC4103590

[eva13283-bib-0011] Brown, D. M., Alphey, L. S., McKemey, A., Beech, C., & James, A. A. (2014). Criteria for identifying and evaluating candidate sites for open‐field trials of genetically engineered mosquitoes. Vector Borne and Zoonotic Diseases, 14(4), 291–299. 10.1089/vbz.2013.1364 24689963 PMC3993056

[eva13283-bib-0012] Campos, E. G., Trevino, H. A., & Strom, L. G. (1961). The dispersal of mosquitoes by railroad trains involved in international traffic. Mosquito News, 21(3), 190–192.

[eva13283-bib-0013] Carballar‐Lejarazú, R., & James, A. A. (2017). Population modification of Anopheline species to control malaria transmission. Pathogens and Global Health, 111(8), 424–435. 10.1080/20477724.2018.1427192 29385893 PMC6066855

[eva13283-bib-0014] Carballar‐Lejarazú, R., Ogaugwu, C., Tushar, T., Kelsey, A., Pham, T. B., Murphy, J., & James, A. A. (2020). Next‐generation gene drive for population modification of the malaria vector mosquito, *Anopheles gambiae* . Proceedings of the National Academy of Sciences, 117(37), 22805–22814. 10.1073/pnas.2010214117 PMC7502704 32839345

[eva13283-bib-0015] Carpenter, S. R. (1996). Microcosm experiments have limited relevance for community and ecosystem ecology. Ecology, 77(3), 677–680. 10.2307/2265490

[eva13283-bib-0016] Cisnetto, V., & Barlow, J. (2020). The development of complex and controversial innovations. Genetically modified mosquitoes for malaria eradication. Research Policy, 49(3), 103917. 10.1016/j.respol.2019.103917 32255861 PMC7104890

[eva13283-bib-0017] Clarkson, C. S., Miles, A., Harding, N. J., O'Reilly, A. O., Weetman, D., Kwiatkowski, D., Donnelly, M. J., & The Anopheles gambiae 1000 Genomes Consortium . (2021). The genetic architecture of target‐site resistance to pyrethroid insecticides in the African malaria vectors *Anopheles gambiae* and *Anopheles coluzzii* . Molecular Ecology. 10.1111/mec.15845 PMC9019111 33590926

[eva13283-bib-0018] Courtier‐Orgogozo, V., Danchin, A., Gouyon, P. H., & Boete, C. (2020). Evaluating the probability of CRISPR‐based gene drive contaminating another species. Evolutionary Applications, 13(8), 1888–1905. 10.1111/eva.12939 32908593 PMC7463340

[eva13283-bib-0019] Dahl, A. L. (1991). Island directory (Prelim. ed.). UNEP.

[eva13283-bib-0020] Danecek, P., Auton, A., Abecasis, G., Albers, C. A., Banks, E., & DePristo, M. A., Handsaker, R. E., Lunter, G., Marth, G. T., Sherry, S. T., McVean, G., & Genomes Project Analysis, G. (2011). The variant call format and VCFtools. Bioinformatics, 27(15), 2156–2158. 10.1093/bioinformatics/btr330 21653522 PMC3137218

[eva13283-bib-0021] Eritja, R., Palmer, J., Roiz, D., Sanpera‐Calbet, I., & Bartumeus, F. (2017). Direct evidence of adult *Aedes albopictus* dispersal by car. Scientific Reports, 7, 14399. 10.1038/s41598-017-12652-5 29070818 PMC5656642

[eva13283-bib-0022] Exmile Solutions Ltd . (2021). Historical AIS data (port calls) for 2019‐01‐01 to 2019‐12‐31 for PORT = BJCOO, CMDLA, TZDAR, CVRAI, CVMIN, ZADUR, KMYVA, KMMUT, Fomboni, STTMS, STPCP.

[eva13283-bib-0023] Facchinelli, L., Valerio, L., Ramsey, J. M., Gould, F., Walsh, R. K., Bond, G., Robert, M. A., Lloyd, A. L., James, A. A., Alphey, L., & Scott, T. W. (2013). Field cage studies and progressive evaluation of genetically‐engineered mosquitoes. PLoS Neglected Tropical Diseases, 7(1), e2001. 10.1371/journal.pntd.0002001 23350003 PMC3547837

[eva13283-bib-0024] Frankham, R. (1997). Do island populations have less genetic variation than mainland populations? Heredity, 78(3), 311–327. 10.1038/hdy.1997.46 9119706

[eva13283-bib-0025] Frean, J., Brooke, B., Thomas, J., & Blumberg, L. (2014). Odyssean malaria outbreaks in Gauteng Province, South Africa, 2007–2013. South African Medical Journal, 104(5), 335–338. 10.7196/SAMJ.7684 25212198

[eva13283-bib-0026] Garrison, E., & Marth, G. (2012). Haplotype‐based variant detection from short‐read sequencing. *ArXiv*, *1207*.

[eva13283-bib-0027] Google Earth (Cartographer) . Map showing location of Kome Island. https://earth.google.com/web/search/Koome+Island,+Uganda/@‐0.08724243,32.74244361,1211.47630539a,48380.8541521d,35y,16.3547808h,11.41826421t,0r/data=CigiJgokCQxDZ9yeljVAEQtDZ9yeljXAGRf30zaOXUJAIW6hBeDnvlDA

[eva13283-bib-0028] Hammond, A., Galizi, R., Kyrou, K., Simoni, A., Siniscalchi, C., Katsanos, D., Gribble, M., Baker, D., Marois, E., Russell, S., Burt, A., Windbichler, N., Crisanti, A., & Nolan, T. (2016). A CRISPR‐Cas9 gene drive system targeting female reproduction in the malaria mosquito vector *Anopheles gambiae* . Nature Biotechnology, 34(1), 78–83. 10.1038/nbt.3439 PMC4913862 26641531

[eva13283-bib-0029] Helmus, M. R., & Behm, J. E. (2020). Island biogeography revisited. In M. I. Goldstein & D. A. DellaSala (Eds.), Encyclopedia of the World's biomes (pp. 51–56). Elsevier.

[eva13283-bib-0030] Helmus, M. R., Mahler, D. L., & Losos, J. B. (2014). Island biogeography of the Anthropocene. Nature, 513(7519), 543–546. 10.1038/nature13739 25254475

[eva13283-bib-0031] Holt, R. A., Subramanian, G. M., Halpern, A., Sutton, G. G., Charlab, R., Nusskern, D. R., & Hoffman, S. L. (2002). The genome sequence of the malaria mosquito *Anopheles gambiae* . Science, 298(5591), 129–149. 10.1126/science.1076181 12364791

[eva13283-bib-0032] Hudson, R. R., Slatkin, M., & Maddison, W. P. (1992). Estimation of levels of gene flow from DNA sequence data. Genetics, 132(2), 583–589. https://www.ncbi.nlm.nih.gov/pubmed/1427045 1427045 10.1093/genetics/132.2.583 PMC1205159

[eva13283-bib-0033] Huestis, D. L., Dao, A., Diallo, M., Sanogo, Z. L., Samake, D., Yaro, A. S., Ousman, Y., Linton, Y.‐M., Krishna, A., Veru, L., Krajacich, B. J., Faiman, R., Florio, J., Chapman, J. W., Reynolds, D. R., Weetman, D., Mitchell, R., Donnelly, M. J., Talamas, E., … Lehmann, T. (2019). Windborne long‐distance migration of malaria mosquitoes in the Sahel. Nature, 574(7778), 404–408. 10.1038/s41586-019-1622-4 31578527 PMC11095661

[eva13283-bib-0034] Idris, Z. M., Chan, C. W., Kongere, J., Gitaka, J., Logedi, J., Omar, A., Obonyo, C., Machini, B. K., Isozumi, R., Teramoto, I., Kimura, M., & Kaneko, A. (2016). High and heterogeneous prevalence of asymptomatic and sub‐microscopic malaria infections on islands in Lake Victoria, Kenya. Scientific Reports, 6, 36958. 10.1038/srep36958 27841361 PMC5107902

[eva13283-bib-0035] Irish, S. R., Kyalo, D., Snow, R. W., & Coetzee, M. (2020). Updated list of Anopheles species (Diptera: Culicidae) by country in the Afrotropical Region and associated islands. Zootaxa, 4747(3), 401–449. 10.11646/zootaxa.4747.3.1 PMC7116328 32230095

[eva13283-bib-0036] Isaacs, A. T., Jasinskiene, N., Tretiakov, M., Thiery, I., Zettor, A., Bourgouin, C., & James, A. A. (2012). Transgenic *Anopheles stephensi* coexpressing single‐chain antibodies resist *Plasmodium falciparum* development. Proceedings of the National Academy of Sciences of the United States of America, 109(28), E1922–E1930. 10.1073/pnas.1207738109 22689959 PMC3396534

[eva13283-bib-0037] Itescu, Y., Foufopoulos, J., Pafilis, P., & Meiri, S. (2020). The diverse nature of island isolation and its effect on land bridge insular faunas. Global Ecology and Biogeography, 29(2), 262–280. 10.1111/geb.13024

[eva13283-bib-0038] Jackson, G., & Gartlan, J. S. (1965). The flora and fauna of Lolui Island, Lake Victoria: A study of vegetation, men and monkeys. Journal of Ecology, 53(3), 573–597. 10.2307/2257622

[eva13283-bib-0039] James, S., Collins, F. H., Welkhoff, P. A., Emerson, C., Godfray, H. C. J., Gottlieb, M., & Toure, Y. T. (2018). Pathway to deployment of gene drive mosquitoes as a potential biocontrol tool for elimination of malaria in Sub‐Saharan Africa: Recommendations of a scientific working group (dagger). American Journal of Tropical Medicine and Hygiene, 98(6_Suppl), 1–49. 10.4269/ajtmh.18-0083 PMC5993454 29882508

[eva13283-bib-0040] James, S. L., Marshall, J. M., Christophides, G. K., Okumu, F. O., & Nolan, T. (2020). Toward the definition of efficacy and safety criteria for advancing gene drive‐modified mosquitoes to field testing. Vector Borne and Zoonotic Diseases, 20(4), 237–251. 10.1089/vbz.2019.2606 32155390 PMC7153640

[eva13283-bib-0041] Japan Aerospace Exploration Agency . (2020). ALOS Global Digital Surface Model (DSM) ALOS World 3D‐30m (AW3D30) Version 3.1. https://www.eorc.jaxa.jp/ALOS/en/aw3d30/aw3d30v31_product_e_a.pdf

[eva13283-bib-0042] Jarvis, A., Reuter, H. I., Nelson, A., Guevara, E. (2008). Hole‐filled seamless SRTM data V4. https://srtm.csi.cgiar.org/

[eva13283-bib-0043] Johnson, K. P., Adler, F. R., & Cherry, J. L. (2000). Genetic and phylogenetic consequences of island biogeography. Evolution, 54(2), 387–396. 10.1111/j.0014-3820.2000.tb00041.x 10937215

[eva13283-bib-0044] Kaufmann, C., & Briegel, H. (2004). Flight performance of the malaria vectors *Anopheles gambiae* and *Anopheles atroparvus* . Journal of Vector Ecology, 29(1), 140–153. 15266751

[eva13283-bib-0045] Kayondo, J. K., Mukwaya, L. G., Stump, A., Michel, A. P., Coulibaly, M. B., Besansky, N. J., & Collins, F. H. (2005). Genetic structure of *Anopheles gambiae* populations on islands in northwestern Lake Victoria, Uganda. Malaria Journal, 4, 59. 10.1186/1475-2875-4-59 16336684 PMC1327676

[eva13283-bib-0046] Knols, B. G. J., & Bossin, H. (2006). Identification and characterization of field sites for genetic control of disease vectors. In B. G. J. Knols & C. Louis (Eds.). Bridging Laboratory and Field Research for Genetic Control of Disease Vectors (pp. 203–209). Springer.

[eva13283-bib-0047] Kolopack, P. A., & Lavery, J. V. (2017). Informed consent in field trials of gene‐drive mosquitoes. Gates Open Research, 1, 14. 10.12688/gatesopenres.12771.1 29355214 PMC5757819

[eva13283-bib-0048] Kormos, A., Lanzaro, G. C., Bier, E., Dimopoulos, G., Marshall, J. M., Pinto, J., Aguiar dos Santos, A., Bacar, A., Sousa Pontes Sacramento Rompão, H., & James, A. A. (2020). Application of the relationship‐based model to engagement for field trials of genetically engineered malaria vectors. American Journal of Tropical Medicine and Hygiene, 104(3), 805–811. 10.4269/ajtmh.20-0868. https://www.ncbi.nlm.nih.gov/pubmed/33350374 PMC7941841 33350374

[eva13283-bib-0049] Kyalo, D., Amratia, P., Mundia, C. W., Mbogo, C. M., Coetzee, M., & Snow, R. W. (2017). A geo‐coded inventory of anophelines in the Afrotropical Region south of the Sahara: 1898–2016. Wellcome Open Research, 2, 1898–2016. 10.12688/wellcomeopenres.12187.1 PMC5558104 28884158

[eva13283-bib-0050] Lee, Y., Marsden, C. D., Norris, L. C., Collier, T. C., Main, B. J., Fofana, A., Cornel, A. J., & Lanzaro, G. C. (2013). Spatiotemporal dynamics of gene flow and hybrid fitness between the M and S forms of the malaria mosquito, *Anopheles gambiae* . Proceedings of the National Academy of Sciences of the United States of America, 110(49), 19854–19859. 10.1073/pnas.1316851110 24248386 PMC3856788

[eva13283-bib-0051] Li, H. (2013). Aligning sequence reads, clone sequences and assembly contigs with BWA‐MEM. *ArXiv, 1303*.

[eva13283-bib-0052] Li, M., Yang, T., Kandul, N. P., Bui, M., Gamez, S., Raban, R., Bennett, J., Sánchez C, H. M., Lanzaro, G. C., Schmidt, H., Lee, Y., Marshall, J. M., & Akbari, O. S. (2020). Development of a confinable gene drive system in the human disease vector *Aedes aegypti* . eLife, 9(1), 1–22. 10.7554/eLife.51701 PMC6974361 31960794

[eva13283-bib-0053] LNRS Data Services Inc . (2021). Q‐00223232 Historical Data Export.

[eva13283-bib-0054] Losos, J. B., & Ricklefs, R. E. (2009). Adaptation and diversification on islands. Nature, 457(7231), 830–836. 10.1038/nature07893 19212401

[eva13283-bib-0055] Lounio, T. (2014). Population Dynamics and Livelihood Change on Ukara Island, Lake Victoria. (Master's Thesis), University of Helsinki.

[eva13283-bib-0056] MacArthur, R. H., & Wilson, E. O. (1967). The theory of island biogeography. Princeton University Press.

[eva13283-bib-0057] Miles, A., & Harding, N. (2017). cggh/scikit‐allel: v1.1.8 (Version v1.1.8). 10.5281/zenodo.822784

[eva13283-bib-0058] Mugono, M., Konje, E., Kuhn, S., Mpogoro, F. J., Morona, D., & Mazigo, H. D. (2014). Intestinal schistosomiasis and geohelminths of Ukara Island, North‐Western Tanzania: Prevalence, intensity of infection and associated risk factors among school children. Parasites & Vectors, 7, 612. 10.1186/s13071-014-0612-5 25533267 PMC4297386

[eva13283-bib-0059] Nambuya, A., Sekwewa, C., Nkwiine, C., Wetala, P. (2013, 20–25 October 2013). Abundance and ecological functional categories of soil macrofauna as indicators of soil chemical properties status in oil palm plantations in Bugala island Kalangala District, Uganda. Paper presented at the Joint Proceedings of 27th Soil Science Society of East Africa & 6th African Soil Science Society, Nakuru, Kenya.

[eva13283-bib-0060] Nampijja, M., Webb, E. L., Kaweesa, J., Kizindo, R., Namutebi, M., Nakazibwe, E., Oduru, G., Kabuubi, P., Kabagenyi, J., Kizito, D., Muhangi, L., Akello, M., Verweij, J. J., Nerima, B., Tukahebwa, E., & Elliott, A. M. (2015). The Lake Victoria Island Intervention Study on Worms and Allergy‐related diseases (LaVIISWA): Study protocol for a randomised controlled trial. Trials, 16, 187. 10.1186/s13063-015-0702-5 25902705 PMC4413531

[eva13283-bib-0061] Nash, A., Urdaneta, G. M., Beaghton, A. K., Hoermann, A., Papathanos, P. A., Christophides, G. K., & Windbichler, N. (2019). Integral gene drives for population replacement. Biology Open, 8(1), bio037762. 10.1242/bio.037762 30498016 PMC6361204

[eva13283-bib-0062] National Academies of Sciences Engineering and Medicine . (2016). Gene drives on the horizon: Advancing science, navigating uncertainty, and aligning research with public values. The National Academies Press. 27536751

[eva13283-bib-0063] Neuhaus, C. P. (2018). Community engagement and field trials of genetically modified insects and animals. Hastings Center Report, 48(1), 25–36. 10.1002/hast.808 29457234

[eva13283-bib-0064] Nieman, C. C., Yamasaki, Y., Collier, T. C., & Lee, Y. (2015). A DNA extraction protocol for improved DNA yield from individual mosquitoes. F1000Research, 4, 1314. 10.12688/f1000research.7413.1 26937269 PMC4743141

[eva13283-bib-0065] Quinlan, A. R., & Hall, I. M. (2010). BEDTools: a flexible suite of utilities for comparing genomic features. Bioinformatics, 26(6), 841–842. 10.1093/bioinformatics/btq033 20110278 PMC2832824

[eva13283-bib-0066] Resnik, D. B. (2014). Ethical issues in field trials of genetically modified disease‐resistant mosquitoes. Developing World Bioethics, 14(1), 37–46. 10.1111/dewb.12011 23279283 PMC3620724

[eva13283-bib-0067] Santos, A. M. C., Field, R., Ricklefs, R. E., & Borregaard, M. (2016). New directions in island biogeography. Global Ecology and Biogeography, 25(7), 751–768. 10.1111/geb.12477

[eva13283-bib-0068] Schindler, D. W. (1998). Whole‐ecosystem experiments: Replication versus realism: The need for ecosystem‐scale experiments. Ecosystems, 1(4), 323–334. 10.1007/s100219900026

[eva13283-bib-0069] Schmidt, H., Lee, Y., Collier, T. C., Hanemaaijer, M. J., Kirstein, O. D., Ouledi, A., Muleba, M., Norris, D. E., Slatkin, M., Cornel, A. J., & Lanzaro, G. C. (2019). Transcontinental dispersal of *Anopheles gambiae* occurred from West African origin via serial founder events. Communications Biology, 2, 473. 10.1038/s42003-019-0717-7 31886413 PMC6923408

[eva13283-bib-0070] Scott, T. W., Takken, W., Knols, B. G., & Boete, C. (2002). The ecology of genetically modified mosquitoes. Science, 298(5591), 117–119. 10.1126/science.298.5591.117 12364785

[eva13283-bib-0071] Service, M. W. (1997). Mosquito (Diptera: Culicidae) dispersal–the long and short of it. Journal of Medical Entomology, 34(6), 579–588. 10.1093/jmedent/34.6.579 9439109

[eva13283-bib-0072] Sharakhova, M. V., Hammond, M. P., Lobo, N. F., Krzywinski, J., Unger, M. F., Hillenmeyer, M. E., & Collins, F. H. (2007). Update of the *Anopheles gambiae* PEST genome assembly. Genome Biology, 8(1), R5. 10.1186/gb-2007-8-1-r5 17210077 PMC1839121

[eva13283-bib-0073] Smith, A. (1955). The transmission of Bancroftial Filariasis on Ukara Island, Tanganyika. I.—A geographical and ecological description of the island with an annotated list of mosquitos and other Arthropods of medical importance. Bulletin of Entomological Research, 46(2), 419–436. 10.1017/S000748530003100X

[eva13283-bib-0074] Ssegawa, P., & Nkuutu, D. N. (2006). Diversity of vascular plants on Ssese islands in Lake Victoria, central Uganda. African Journal of Ecology, 44(1), 22–29. 10.1111/j.1365-2028.2006.00609.x

[eva13283-bib-0075] Tan, A., Abecasis, G. R., & Kang, H. M. (2015). Unified representation of genetic variants. Bioinformatics, 31(13), 2202–2204. 10.1093/bioinformatics/btv112 25701572 PMC4481842

[eva13283-bib-0076] Tarasov, A., Vilella, A. J., Cuppen, E., Nijman, I. J., & Prins, P. (2015). Sambamba: Fast processing of NGS alignment formats. Bioinformatics, 31(12), 2032–2034. 10.1093/bioinformatics/btv098 25697820 PMC4765878

[eva13283-bib-0077] Tuhebwe, D., Bagonza, J., Kiracho, E. E., Yeka, A., Elliott, A. M., & Nuwaha, F. (2015). Uptake of mass drug administration programme for schistosomiasis control in Koome Islands, Central Uganda. PLoS One, 10(4), e0123673. 10.1371/journal.pone.0123673 25830917 PMC4382187

[eva13283-bib-0078] Warren, B. H., Simberloff, D., Ricklefs, R. E., Aguilée, R., Condamine, F. L., Gravel, D., Morlon, H., Mouquet, N., Rosindell, J., Casquet, J., Conti, E., Cornuault, J., Fernández‐Palacios, J. M., Hengl, T., Norder, S. J., Rijsdijk, K. F., Sanmartín, I., Strasberg, D., Triantis, K. A., … Thébaud, C. (2015). Islands as model systems in ecology and evolution: Prospects fifty years after MacArthur‐Wilson. Ecology Letters, 18(2), 200–217. 10.1111/ele.12398 25560682

[eva13283-bib-0079] Weigelt, P., Jetz, W., & Kreft, H. (2013). Bioclimatic and physical characterization of the world’s islands. Proceedings of the National Academy of Sciences, 110(38), 15307–15312. 10.1073/pnas.1306309110 PMC3780862 24003123

[eva13283-bib-0080] White, G. B. (1971). Chromosomal evidence for natural interspecific hybridization by mosquitoes of the *Anopheles gambiae* complex. Nature, 231(5299), 184–185. 10.1038/231184a0 4930676

[eva13283-bib-0081] WHO/TDR and FNIH . (2014). The Guidance Framework for testing genetically modified mosquitoes [Framework]. https://www.who.int/tdr/publications/year/2014/Guidance_framework_mosquitoes.pdf

[eva13283-bib-0082] Wynn, G., & Paradise, C. J. (2001). Effects of microcosm scaling and food resources on growth and survival of larval *Culex pipiens* . BMC Ecology, 1, 3. 10.1186/1472-6785-1-3 11527507 PMC45585

[eva13283-bib-0083] Yamasaki, Y. K., Nieman, C. C., Chang, A. N., Collier, T. C., Main, B. J., & Lee, Y. (2016). Improved tools for genomic DNA library construction of small insects [version 1; not peer reviewed]. F1000Research, 5, 211 (poster). 10.7490/f1000research.1111322.1

[eva13283-bib-0084] Zeemeijer, I. M. (2012). Who gets What, When and How? New Corporate Land Acquisitions and the Impact on Local Livelihoods in Uganda. (Master's Thesis), Utrecht University.

