# Supplementary figures and images for: Selection of sites for field trials of genetically engineered mosquitoes with gene drive
Source: Evol Appl. 2021 Aug 10;14(9):2147–61. doi: 10.1111/eva.13283 (PMC8477601; doi:10.1111/eva.13283)

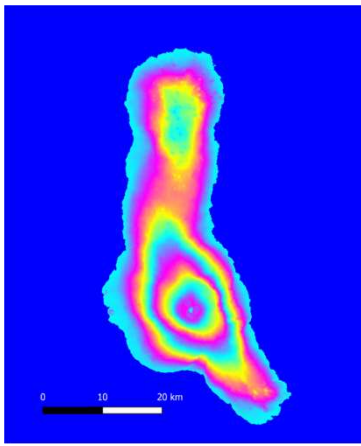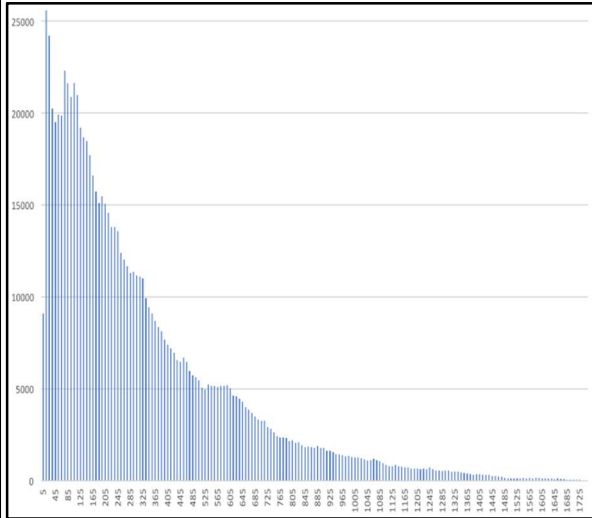

**(a) Grande Comore topography**

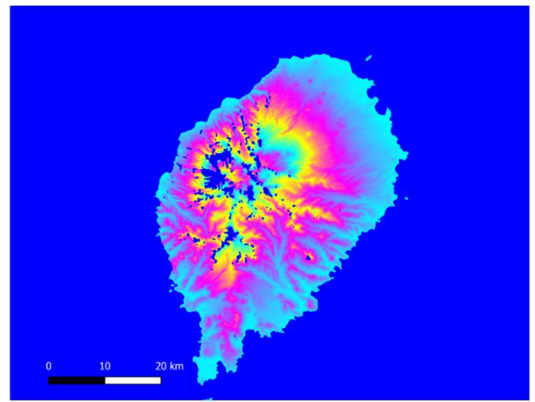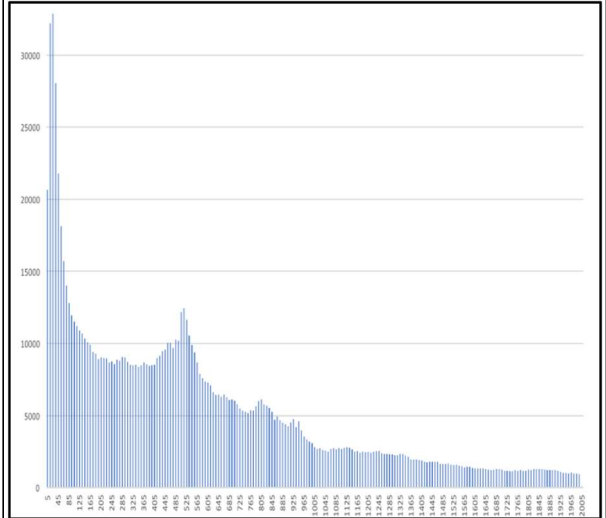

**(b) São Tomé topography**

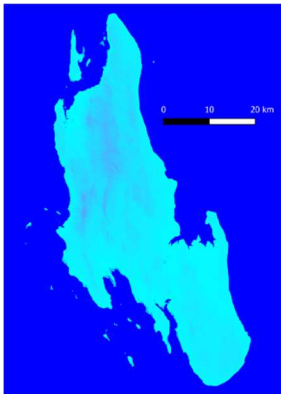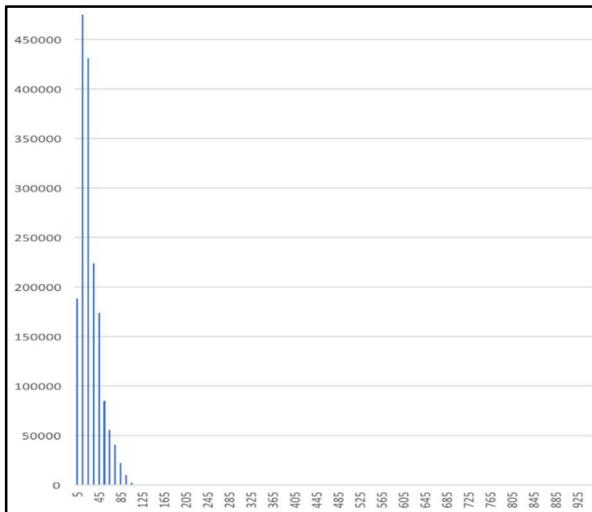

**(c) Zanzibar topography**

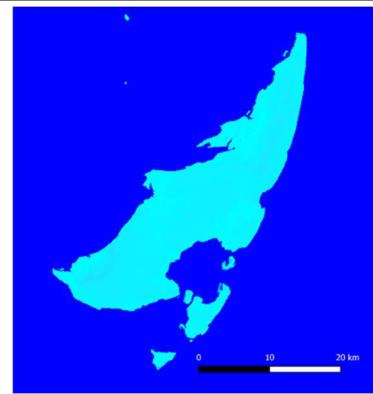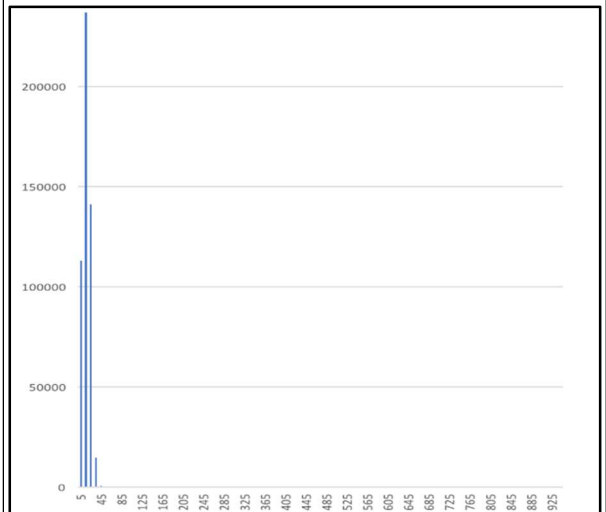

**(d) Mafia topography**

Supplement: Supplementary file 1 — Fig S1 [file EVA-14-2147-s006.pdf]

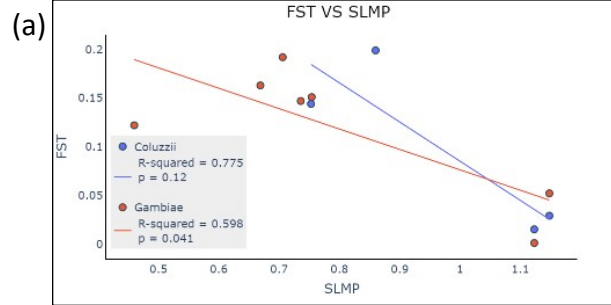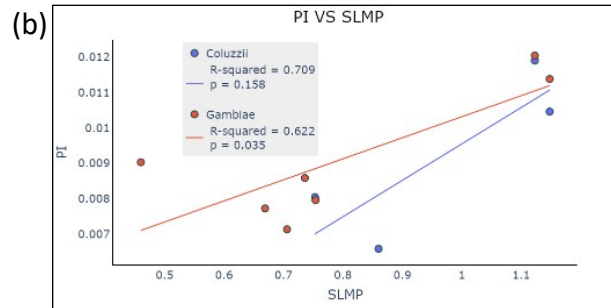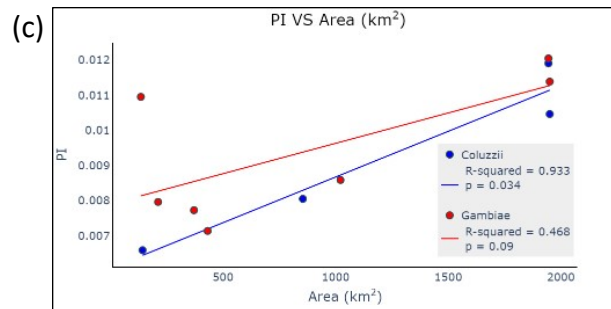

Supplement: Supplementary file 3 — Fig S3 [file EVA-14-2147-s004.pdf]
