# Supplementary material for: Selection of sites for field trials of genetically engineered mosquitoes with gene drive
Source: Evol Appl. 2021 Aug 10;14(9):2147–61. doi: 10.1111/eva.13283 (PMC8477601; doi:10.1111/eva.13283)
Supplement: Supplementary file 2 — Fig S2 [file EVA-14-2147-s001.pdf]

(a) ***Anopheles gambiae*** populations

		Zambia	Uganda	The Gambia*	Tanzania	Mali	Guinea-Bissau*	Guinea	Ghana	Gabon	Cameroon	Burkina Faso	Madagascar	Mohéli	Mayotte	Grande Comore	Anjouan	Sserinya	Nsadzi	Bukasa	Bugala	Banda	Bioko
Island	Formosa	0.079	0.062	0.012	0.137	0.049	0.001	0.058	0.060	0.093	0.059	0.058	0.167	0.192	0.210	0.182	0.227	0.072	0.075	0.074	0.063	0.085	0.079
Island	Bioko	0.062	0.041	0.073	0.120	0.031	0.083	0.034	0.040	0.069	0.034	0.036	0.153	0.180	0.200	0.168	0.220	0.051	0.055	0.055	0.043	0.067	
Island	Banda	0.042	0.029	0.078	0.099	0.037	0.089	0.042	0.046	0.075	0.041	0.043	0.133	0.162	0.180	0.146	0.203	0.020	0.029	0.028	0.022		
Island	Bugala	0.024	0.003	0.055	0.081	0.014	0.066	0.016	0.022	0.052	0.016	0.018	0.118	0.146	0.164	0.130	0.186	0.010	0.014	0.012			
Island	Bukasa	0.027	0.020	0.067	0.084	0.027	0.077	0.031	0.036	0.063	0.030	0.032	0.119	0.148	0.166	0.132	0.188	0.019	0.020				
Island	Nsadzi	0.033	0.019	0.067	0.088	0.027	0.077	0.031	0.037	0.064	0.031	0.032	0.122	0.153	0.171	0.134	0.192	0.019					
Island	Sserinya	0.031	0.014	0.064	0.087	0.023	0.075	0.026	0.031	0.060	0.025	0.028	0.123	0.152	0.170	0.136	0.194						
Island	Anjouan	0.164	0.195	0.224	0.191	0.182	0.230	0.204	0.208	0.223	0.204	0.204	0.193	0.137	0.174	0.201							
Island	Grande Comore	0.108	0.138	0.176	0.130	0.134	0.184	0.149	0.154	0.169	0.148	0.150	0.163	0.166	0.196								
Island	Mayotte	0.142	0.173	0.204	0.169	0.164	0.212	0.182	0.185	0.200	0.182	0.182	0.157	0.126									
Island	Mohéli	0.122	0.154	0.187	0.148	0.147	0.194	0.164	0.168	0.181	0.163	0.164	0.153										
Island	Madagascar	0.093	0.126	0.161	0.122	0.122	0.169	0.136	0.139	0.154	0.135	0.136											
Mainland	Burkina Faso	0.043	0.016	0.050	0.101	0.002	0.063	0.000	0.008	0.052	0.003												
Mainland	Cameroon	0.042	0.013	0.051	0.099	0.002	0.063	0.002	0.009	0.050													
Mainland	Gabon	0.062	0.053	0.086	0.121	0.046	0.096	0.050	0.056														
Mainland	Ghana	0.047	0.019	0.053	0.105	0.007	0.065	0.007															
Mainland	Guinea	0.043	0.015	0.049	0.100	0.001	0.062																
Mainland	Guinea-Bissau*	0.081	0.066	0.011	0.140	0.052																	
Mainland	Mali	0.036	0.012	0.041	0.090																		
Mainland	Tanzania	0.059	0.090	0.131																			
Mainland	The Gambia*	0.072	0.055																				
Mainland	Uganda	0.033																					

	Mean F_{ST} between Neighboring	
	Nearest Mainland	Nearest Island
Formosa	0.001	-
Bioko	0.052	-
Banda	0.029	0.025
Bugala	0.003	0.014
Bukasa	0.020	0.020
Nsadzi	0.019	0.021
Sserinya	0.014	0.017
Anjouan	0.192	0.171
Grande Comore	0.147	0.188
Mayotte	0.163	0.165
Mohéli	0.151	0.143
Madagascar	0.122	0.167

(b) ***Anopheles coluzzii*** populations

		The Gambia*	Mali	Guinea-Bissau*	Guinea	Ghana	Gabon	Cote d'Ivoire	Cameroon	Burkina Faso	Benin	Angola	São Tomé	Príncipe	Bioko
Island	Formosa	0.030	0.028	0.015	0.053	0.032	0.088	0.031	0.076	0.032	0.037	0.133	0.176	0.225	0.090
Island	Bioko	0.108	0.072	0.107	0.110	0.073	0.036	0.076	0.022	0.076	0.078	0.094	0.151	0.207	
Island	Príncipe	0.238	0.212	0.238	0.250	0.219	0.204	0.220	0.195	0.220	0.223	0.227	0.130		
Island	São Tomé	0.191	0.161	0.190	0.200	0.167	0.148	0.168	0.139	0.168	0.171	0.168			
Mainland	Angola	0.149	0.115	0.148	0.156	0.122	0.082	0.123	0.080	0.121	0.126				
Mainland	Benin	0.065	0.017	0.062	0.054	0.009	0.075	0.014	0.064	0.019					
Mainland	Burkina Faso	0.061	0.001	0.060	0.056	0.018	0.072	0.015	0.062						
Mainland	Cameroon	0.095	0.057	0.094	0.096	0.060	0.024	0.061							
Mainland	Cote d'Ivoire	0.059	0.014	0.054	0.045	0.008	0.074								
Mainland	Gabon	0.108	0.068	0.106	0.108	0.071									
Mainland	Ghana	0.061	0.016	0.057	0.048										
Mainland	Guinea	0.079	0.051	0.071											
Mainland	Guinea-Bissau*	0.011	0.053												
Mainland	Mali	0.055													

	Mean F_{ST} between Neighboring	
	Nearest Mainland	Nearest Island
Formosa	0.015	-
Bioko	0.029	0.207
Príncipe	0.199	0.130
São Tomé	0.144	0.130
