# Supplementary material for: Selection of sites for field trials of genetically engineered mosquitoes with gene drive
Source: Evol Appl. 2021 Aug 10;14(9):2147–61. doi: 10.1111/eva.13283 (PMC8477601; doi:10.1111/eva.13283)
Supplement: Supplementary file 6 — Table S2 [file EVA-14-2147-s009.pdf]

Supplemental Table 2. Comparison of mainland versus island *Anopheles* species diversity and malaria incidence.

MAINLAND

Country	Primary Vectors	Secondary Vectors	Other *Anopheles* (non-vector or status unclear)	References	Malaria Cases (per year)
Burkina Faso	*An. arabiensis, coluzzii, funestus, gambiae, nili*	*An. brunnipes, coustani, cydippis, hancocki, leesoni, maculipalpis, paludis, pharoensis, rivulorum, rufipes, pretoriensis, sergentii, squamosus, theileri, ziemanni*	*An. argenteolobatus, brohieri, brumpti, domicolus, dureni, flavicosta, freetownensis, implexus, longipalpis, murphyi, natalensis, obscurus, rhodesiensis, somalicus, wellcomei*	1,2	6,840,864 (2000)<br>8,602,187 (2010)<br>7,245,827 (2015)<br>7,859,000 (2019)
Cameroon	*An. arabiensis, coluzzii, funestus, gambiae, moucheti, nili*	An. *bervoetsi, brunnipes, carnevalei, coustani, cydippis, demeilloni, hancocki, leesoni, maculipalpis, marshallii, melas, obscurus, ovengensis, paludis, pharoensis, pretoriensis, rivulorum, rivulorum*-like, *rufipes, sergentii, squamosus, ziemanni*	*An. brohieri, buxtoni, christyi, cinctus, concolor, deemingi, domicolus, dualaensis, eouzani, flavicosta, freetownensis, hargreavesi, implexus, jebudensis, longipalpis, mousinhoi, multicinctus, namibiensis, natalensis, okuensis, rageaui, rhodesiensis, smithii, somalicus, tenebrosus, wellcomei*		6,291,500 (2000)<br>5,909,335 (2010)<br>5,777,768 (2015)<br>6,291,256 (2019)
Mali	*An. arabiensis, coluzzii, funestus, gambiae, nili*	*An. brunnipes, coustani, dthali, hancocki, leesoni, maculipalpis, paludis, pharoensis, pretoriensis, rivulorum, rufipes, sergentii, squamosus, ziemanni*	*An. brohieri, domicolus, flavicosta, obscurus, rhodesiensis, somalicus, wellcomei*		4,446,769 (2000)<br>5,772,983 (2010)<br>6,833,022 (2015)<br>6,560,000 (2019)
Tanzania	*An. arabiensis, funestus, gambiae, moucheti, nili*	*An. aruni, brunnipes, cinereus, coustani, cydippis, demeilloni, gibbinsi, leesoni, maculipalpis, marshallii, merus, paludis, parensis, pharoensis, pretoriensis, quadriannulatus, rivulorum, rufipes, squamosus, theileri, ziemanni*	*An. ardensis, argenteolobatus, christyi, confusus, distinctus, erepens, garnhami, implexus, keniensis, kingi, letabensis, longipalpis, lovettae, machardyi, namibiensis, natalensis, njombiensis, rhodesiensis, schwetzi, seydeli, swahilicus, tenebrosus, walravensi, wellcomei, wilsoni*		11,514,222 (2000)<br>5,917,848 (2010)<br>7,298,719 (2015)<br>6,453,096 (2019)
Uganda	*An. arabiensis, funestus, gambiae, moucheti, nili*	*An. bwambae, cinereus, coustani, cydippis, demeilloni, gibbinsi, hancocki, leesoni, maculipalpis, marshallii, paludis, parensis, pharoensis, pretoriensis, quadriannulatus, rivulorum, rufipes, squamosus, symesi, theileri, ziemanni*	*An. ardensis, brohieri, christyi, domicolus, garnhami, hargreavesi, harperi, implexus, keniensis, kingi, longipalpis, natalensis, obscurus, rhodesiensis, tenebrosus, vinckei, wellcomei*		11,522,961 (2000)<br>13,277,279 (2010)<br>9,690,714 (2015)<br>11,629,246 (2019)

ISLANDS

Island	Primary Vectors	Secondary Vectors	Other *Anopheles* (non-vector or status unclear)		Malaria Cases (year)
Bijagos Islands (Guinea-Bissau)	*An. arabiensis, coluzzii, gambiae*	*An. melas,* ***cinereus, coustani, maculipalpis, pharoensis, rufipes, squamosus, ziemanni***	***An. dancailicus, hargreavesi, smithii***	3, 4	
Bioko (Equatorial Guinea)	*An. coluzzii, funestus, gambiae,* ***moucheti***	***An. brunnipes, carnevalei, leesoni, melas, ovengensis***	***An. cinctus, lloreti, obscurus, smithii***	5, 6	
Canary Islands (Spain)		*An.multicolor, sergentii*	*An.hispaniola* (historically a vector in Europe)	7	
Cape Verde	*An. arabiensis*	*An. pretoriensis*		8	144 (2000)<br>47 (2010)<br>7 (2015)<br>0 (2019)
Anjouan (Comoros)	*An. funestus, gambiae*	*An. coustani, mascarensis, pretoriensis*		9, 10, 11	35,309 (2000)<br>36,538 (2010)<br>1,300 (2015)<br>17,599 (2019)
Grand Comore	*An. gambiae*	*An. pretoriensis*			

(Comoros)					
Moheli (Comoros)	*An. funestus, gambiae*	*An. coustani, maculipalpis, mascarensis, pretoriensis*			
Lake Victoria islands	*An. arabiensis, funestus, gambiae*	*An. coustani, pharoensis, symesi, ziemanni*		12, 13, 14	
Mayotte (France)	*An. funestus, gambiae*	*An. coustani, maculipalpis, mascarensis, pretoriensis*	*An. comorensis*	1	>2,000 (2000)<br>433 (2010)<br>14 (2015)<br>22 (2016)
Île Europa (France)	*An. gambiae*			15	
Madagascar	*An. arabiensis, funestus, gambiae, coustani*	*An. brunnipes, cydippis, maculipalpis, mascarensis, merus, pharoensis, pretoriensis, rufipes, squamosus*	*An. flavicosta, fuscicolor, grassei, grenieri, griveaudi, lacani, milloti, notleyi, pauliani, radama, ranci, roubaudi, tenebrosus*	1	901,335 (2000)<br>893,540 (2010)<br>1,897,533 (2015)<br>2,052,071 (2019)
Pemba (Tanzania)	*An. arabiensis, gambiae*	*An. merus*		17	
Príncipe	*An. coluzzii*			18, 19	31,975 (2000)<br>2,740 (2010)<br>2,058 (2015)<br>2,446 (2019)
São Tomé	*An. coluzzii, funestus, gambiae*	*An. coustani, melas, paludis, pharoensis*			
Annobón	*An. coluzzii*			20	
Mauritius	*An. arabiensis*	*An. coustani, maculipalpis, merus*		21	All imported:<br>52 (2010)<br>33 (2012)
Réunion (France)	*An. arabiensis*	*An. coustani*		22	Eliminated (1979)
Zanzibar (Tanzania)	*An. arabiensis, funestus, gambiae*	*An. aruni, coustani, leesoni, maculipalpis, marshallii, merus, paludis, parensis, pretoriensis, rivulorum, squamosus, ziemanni*	*An. longipalpis, obscurus, quadriannulatus, swahilicus, tenebrosus, wellcomei*	2	3,528 (2005)<br>2,572 (2012)<br>3,814 (2015)<br>3,025 (2016)

TABLE 2 REFS:

[1] Irish, S. R., Kyalo, D., Snow, R. W., & Coetzee, M. (2020). Updated list of Anopheles species (Diptera: Culicidae) by country in the Afrotropical Region and associated islands. Zootaxa, 4747(3), zootaxa 4747 4743 4741. Retrieved from https://www.ncbi.nlm.nih.gov/pubmed/32230095. doi:10.11646/zootaxa.4747.3.1

[2] Kyalo, D., Amratia, P., Mundia, C. W., Mbogo, C. M., Coetzee, M., & Snow, R. W. (2017). A geo-coded inventory of anophelines in the Afrotropical Region south of the Sahara: 1898-2016. Wellcome Open Res, 2, 57. Retrieved from https://www.ncbi.nlm.nih.gov/pubmed/28884158. doi:10.12688/wellcomeopenres.12187.1

[3] Ant, T., Foley, E., Tytheridge, S., Johnston, C., Goncalves, A., Ceesay, S., et al. (2020). A survey of Anopheles species composition and insecticide resistance on the island of Bubaque, Bijagos Archipelago, Guinea-Bissau. *Malar J, 19*(1), 27. Retrieved from https://www.ncbi.nlm.nih.gov/pubmed/31941507. doi:10.1186/s12936-020-3115-1

[4] Sanford, M. R., Cornel, A. J., Nieman, C. C., Dinis, J., Marsden, C. D., Weakley, A. M., et al. (2014). Plasmodium falciparum infection rates for some Anopheles spp. from Guinea-Bissau, West Africa. *F1000Res, 3*, 243. Retrieved from https://www.ncbi.nlm.nih.gov/pubmed/25383188. doi:10.12688/f1000research.5485.2

[5] Berzosa, P. J., Cano, J., Roche, J., Rubio, J. M., Garcia, L., Moyano, E., et al. (2002). Malaria vectors in Bioko Island (Equatorial Guinea): PCR determination of the members of Anopheles gambiae Giles complex (Diptera: Culicidae) and pyrethroid knockdown resistance (kdr) in An. gambiae sensu stricto. *J Vector Ecol, 27*(1), 102-106. Retrieved from https://www.ncbi.nlm.nih.gov/pubmed/12125862.

[6] Guerra, C., Fuseini, G., Donfack, O., Smith, J., Mifumu, T., Akadiri, G., et al. (2020). Malaria outbreak in Riaba district, Bioko Island: lessons learned. *Malaria Journal, 19*. doi:10.1186/s12936-020-03347-w

[7] Baez, M., & Fernandez, J. M. (1980). Notes on the mosquito fauna of the Canary Islands (Diptera: Culicidae) *Mosquito Systematics, 12*(3), 349-355.

[8] Alves, J., Gomes, B., Rodrigues, R., Silva, J., Arez, A. P., Pinto, J., et al. (2010). Mosquito fauna on the Cape Verde Islands (West Africa): an update on species distribution and a new finding. *J Vector Ecol, 35*(2), 307-312. Retrieved from https://www.ncbi.nlm.nih.gov/pubmed/21175936. doi:10.1111/j.1948-7134.2010.00087.x

[9] Brunhes, J. (1977). Les moustiques de l'archipel des Comores. *Cahiers ORSTOM. Série Entomologie Médicale et Parasitologie, 25*(2), 131-152.

[10]Brunhes, J., Le Goff, G., & Geoffroy, B. (1997). Anophèles afro-tropicaux : 1. Descriptions d'espèces nouvelles et changements de statuts taxonomiques (Diptera : Culicidae). *Annales de la Societe Entomologique de France, 33*.

[11] Coetzee, M., Hunt, R. H., Wilkerson, R., Della Torre, A., Coulibaly, M. B., & Besansky, N. J. (2013). Anopheles coluzzii and Anopheles amharicus, new members of the Anopheles gambiae complex. *Zootaxa, 3619*, 246-274. Retrieved from https://www.ncbi.nlm.nih.gov/pubmed/26131476.

[12] Ajamma, Y. U., Villinger, J., Omondi, D., Salifu, D., Onchuru, T. O., Njoroge, L., et al. (2016). Composition and Genetic Diversity of Mosquitoes (Diptera: Culicidae) on Islands and Mainland Shores of Kenya's Lakes Victoria and Baringo. *Journal of Medical Entomology, 53*(6), 1348-1363. Retrieved from https://www.ncbi.nlm.nih.gov/pubmed/27402888. doi:10.1093/jme/tjw102

[13] Lukindu, M., Bergey, C. M., Wiltshire, R. M., Small, S. T., Bourke, B. P., Kayondo, J. K., et al. (2018). Spatio-temporal genetic structure of Anopheles gambiae in the Northwestern Lake Victoria Basin, Uganda: implications for genetic control trials in malaria endemic regions. *Parasit Vectors, 11*(1), 246. Retrieved from https://www.ncbi.nlm.nih.gov/pubmed/29661226. doi:10.1186/s13071-018-2826-4

[14] Ogola, E., Villinger, J., Mabuka, D., Omondi, D., Orindi, B., Mutunga, J., et al. (2017). Composition of Anopheles mosquitoes, their blood-meal hosts, and Plasmodium falciparum infection rates in three islands with disparate bed net coverage in Lake Victoria, Kenya. *Malar J, 16*(1), 360. Retrieved from https://www.ncbi.nlm.nih.gov/pubmed/28886724. doi:10.1186/s12936-017-2015-5

[15] Boussès, P., Dehecq, J. S., Brengues, C., & Fontenille, D. (2013). Inventaire actualisé des moustiques (Diptera : Culicidae) de l'île de La Réunion, océan Indien. *Bulletin de la Société de pathologie exotique, 106*(2), 113-125. Retrieved from https://doi.org/10.1007/s13149-013-0288-7. doi:10.1007/s13149-013-0288-7

[16] World Health Organization. (2020). *World malaria report 2020: 20 years of global progress and challenges*. Retrieved from Geneva:

[17] Haji, K. A., Khatib, B. O., Smith, S., Ali, A. S., Devine, G. J., Coetzee, M., et al. (2013). Challenges for malaria elimination in Zanzibar: pyrethroid resistance in malaria vectors and poor performance of long-lasting insecticide nets. *Parasit Vectors, 6*, 82. Retrieved from https://www.ncbi.nlm.nih.gov/pubmed/23537463. doi:10.1186/1756-3305-6-82

[18] Campos, M., Hanemaaijer, M., Gripkey, H., Collier, T., Lee, Y., Cornel, A., et al. (2021). *The origin of island populations of the African malaria mosquito, Anopheles coluzzii*: Communications Biology, in press.

[19] Loiseau, C., Melo, M., Lee, Y., Pereira, H., Hanemaaijer, M. J., Lanzaro, G. C., et al. (2019). High endemism of mosquitoes on São Tomé and Príncipe Islands: evaluating the general dynamic model in a worldwide island comparison. *Insect Conservation and Diversity, 12*(1), 69-79. Retrieved from https://onlinelibrary.wiley.com/doi/abs/10.1111/icad.12308. doi:https://doi.org/10.1111/icad.12308

[20] Salgueiro, P., Moreno, M., Simard, F., O'Brochta, D., & Pinto, J. (2013). New insights into the population structure of Anopheles gambiae s.s. in the Gulf of Guinea Islands revealed by Herves transposable elements. *PLoS One, 8*(4), e62964. Retrieved from https://www.ncbi.nlm.nih.gov/pubmed/23638171. doi:10.1371/journal.pone.0062964

[21] Iyaloo, D. P., Elahee, K. B., Bheecarry, A., & Lees, R. S. (2014). Guidelines to site selection for population surveillance and mosquito control trials: a case study from Mauritius. *Acta Tropica, 132 Suppl*, S140-149. Retrieved from https://www.ncbi.nlm.nih.gov/pubmed/24280144. doi:10.1016/j.actatropica.2013.11.011

[22] WHO Expert Committee on Malaria & World Health Organization. (1979). *WHO Expert Committee on Malaria : seventeenth report* (9241206403). Retrieved from Geneva: https://apps.who.int/iris/handle/10665/41359
