# Supplementary material for: Selection of sites for field trials of genetically engineered mosquitoes with gene drive
Source: Evol Appl. 2021 Aug 10;14(9):2147–61. doi: 10.1111/eva.13283 (PMC8477601; doi:10.1111/eva.13283)
Supplement: Supplementary file 8 — Table S4 [file EVA-14-2147-s008.pdf]

Supplemental Table 4. Outgoing ship traffic and destinations.

PORT	COUNTRY	DESTINATION							
		Africa	Asia	Australia	Europe	Middle East	N. America	Russia	S. America
COTONOU	Benin	959	13	0	921	5	29	4	351
DAR ES SALAAM	Tanzania	2606	264	2	4	94	5	0	14
DOUALA	Cameroon	1796	438	0	150	1	122	0	361
DURBAN	South Africa	2553	580	54	135	210	219	1	297
MINDELO	Cape Verde	1771	23	2	750	6	613	6	362
PRAIA	Cape Verde	1162	0	4	33	0	13	0	32
FOMBONI	Comoros	2	0	0	0	0	0	0	0
MORONI	Comoros	326	1	0	0	14	9	0	0
MUTSAMUDU	Comoros	588	0	0	0	18	0	0	0
PRINCIPE	Sao Tome and Principe	23	0	0	0	0	0	0	0
SAO TOME	Sao Tome and Principe	28	1	0	21	0	3	0	3
